# Skeletal muscle and plasma lipidomic signatures of insulin resistance and overweight/obesity in humans

**DOI:** 10.1002/oby.21448

**Published:** 2016-02-24

**Authors:** Katherine T. Tonks, Adelle CF Coster, Michael J. Christopher, Rima Chaudhuri, Aimin Xu, Johann Gagnon‐Bartsch, Donald J. Chisholm, David E. James, Peter J. Meikle, Jerry R. Greenfield, Dorit Samocha‐Bonet

**Affiliations:** ^1^ Diabetes and Metabolism Division Garvan Institute of Medical Research Sydney New South Wales Australia; ^2^ Faculty of Medicine UNSW Sydney New South Wales Australia; ^3^ Department of Endocrinology and Diabetes Centre St. Vincent's Hospital Sydney New South Wales Australia; ^4^ School of Mathematics and Statistics UNSW Sydney New South Wales Australia; ^5^ Baker IDI Heart and Diabetes Institute Melbourne Victoria Australia; ^6^ Charles Perkins Centre, School of Molecular Bioscience, School of Medicine University of Sydney Sydney New South Wales Australia; ^7^ Department of Medicine, Department of Pharmacology and Pharmacy, and Research Center of Heart, Brain, Hormone and Healthy Ageing University of Hong Kong Pokfulam Hong Kong; ^8^ Department of Statistics University of Michigan Ann Arbor, Michigan USA

## Abstract

**Objective:**

Alterations in lipids in muscle and plasma have been documented in insulin‐resistant people with obesity. Whether these lipid alterations are a reflection of insulin resistance or obesity remains unclear.

**Methods:**

Nondiabetic sedentary individuals not treated with lipid‐lowering medications were studied (*n* = 51). Subjects with body mass index (BMI) > 25 kg/m^2^ (*n* = 28) were stratified based on median glucose infusion rate during a hyperinsulinemic‐euglycemic clamp into insulin‐sensitive and insulin‐resistant groups (above and below median, obesity/insulin‐sensitive and obesity/insulin‐resistant, respectively). Lean individuals (*n* = 23) served as a reference group. Lipidomics was performed in muscle and plasma by liquid chromatography electrospray ionization‐tandem mass spectrometry. Pathway analysis of gene array in muscle was performed in a subset (*n* = 35).

**Results:**

In muscle, insulin resistance was characterized by higher levels of C18:0 sphingolipids, while in plasma, higher levels of diacylglycerol and cholesterol ester, and lower levels of lysophosphatidylcholine and lysoalkylphosphatidylcholine, indicated insulin resistance, irrespective of overweight/obesity. The sphingolipid metabolism gene pathway was upregulated in muscle in insulin resistance independent of obesity. An overweight/obesity lipidomic signature was only apparent in plasma, predominated by higher triacylglycerol and lower plasmalogen species.

**Conclusions:**

Muscle C18:0 sphingolipids may play a role in insulin resistance independent of excess adiposity.

## Introduction

Overweight and obesity are major risk factors of type 2 diabetes, but some people with obesity may be relatively protected from developing type 2 diabetes. The key determinant of “metabolic health” in obesity is insulin sensitivity 1. However, the majority of the population with obesity is insulin‐resistant. Numerous factors have been implicated in the etiology of insulin resistance in obesity, including increased visceral adiposity, liver lipids, impaired adipose tissue expansion, and adipose tissue inflammation 1.

Alterations in skeletal muscle lipids have received much attention in the study of insulin resistance. Most studies have found increased triacylglycerol (TG) in skeletal muscle in sedentary insulin‐resistant individuals, but increased TG in muscle in insulin‐sensitive women and athletes suggests TG are metabolically inactive 2. Conversely, diacylglycerol (DG) and ceramide (Cer) have been implicated in insulin resistance in animal models. Cer is the “hub” lipid in sphingolipid metabolism, serving as a precursor for sphingomyelin (SM) and the higher order sphingolipids monohexosylceramide (MHC/HexCer), dihexosylceramide (DHC/Hex2Cer), trihexosylcermide (THC/Hex3Cer), and G_M3_ ganglioside (GM3) and generated *de novo* from dihydroceramide (dhCer) or breakdown of SM 2, 3. Studies in various animal models, including from our group, have identified Cer(d18:1/16:0) and/or Cer(d18:1/18:0) in liver 4, 5, 6 and skeletal muscle 5 as potential players in insulin resistance. In humans, the contribution of Cer and DG to insulin resistance is still debated. Muscle DG(18:0_20:4), DG(16:0_16:0), and DG(18:0_18:0) were increased in type 2 diabetes compared with nondiabetic individuals with similar obesity and lean athletes 7, and muscle Cer(d18:1/16:0) and Cer(d18:1/18:0) were increased in insulin‐resistant versus insulin‐sensitive women with obesity 8. However, others have found neither DG 2, 5, 13 nor Cer 9, 10 alterations in muscle in insulin‐resistant individuals.

Circulating lipid species likely represent a readout of tissues, particularly liver 11, and possibly also reflect muscle lipid composition. Our studies have revealed that the plasma lipidome signature explained much of the variability in glucose homeostasis in large cohorts 12, and specific lipids emerged as potential biomarkers of glucose intolerance and type 2 diabetes, including Cer(d18:1/18:0) and its dhCer precursor 13.

While large cohort studies are powerful in identifying potential lipid contributors to metabolic disease, a major limitation is the inability to distinguish between lipid correlates of insulin resistance and obesity. Here we report a comprehensive lipidomic analysis of skeletal muscle and plasma in adiposity‐matched insulin‐resistant and insulin‐sensitive individuals compared to a lean insulin‐sensitive group. We use hyperinsulinemic‐euglycemic clamps to define insulin sensitivity and microarray‐based gene expression (GE) to uncover differences in metabolic pathway regulation in muscle. This study design enabled dissection of tissue lipids and lipid‐related pathways potentially involved in insulin resistance from those associated with obesity *per se* in humans.

## Methods

### Participants

Eighty‐one individuals aged 40‐70 years were included in the original study 14. For lipidomic analyses, type 2 diabetes patients (*n* = 21) and individuals treated with lipid‐lowering medications (statins [*n* = 8] or ezetimibe [*n* = 1]) were excluded; therefore, findings for *n* = 51 are reported. Inclusion and exclusion criteria were reported previously 14. The study was approved by the Human Research and Ethics Committee, St. Vincent's Hospital, Sydney, and participants provided informed written consent before study commencement.

### Definition of insulin‐sensitive and insulin‐resistant individuals with overweight/obesity

Individuals with body mass index (BMI) > 25 kg/m^2^ (*n* = 28) were stratified based on median glucose infusion rate (GIR) normalized to fat‐free mass (FFM) with separate cutoffs for men and women (52 and 93 µmol/min/kgFFM, respectively), into insulin‐sensitive (OIS, ≥median, *n* = 14) and insulin‐resistant (OIR, <median, *n* = 14) groups.

### Metabolic assessment

Hyperinsulinemic (80 mU/m^2^/min)‐euglycemic (5 mmol/L) 2.5‐h clamps with indirect calorimetry (ParvoMedics Inc. UT, US) at baseline and clamp steady state to determine resting and insulin‐stimulated respiratory quotient (RQ) were performed, as described 14. Vastus lateralis muscle was biopsied at baseline and 30 and 145 min of the clamp, snap‐frozen and stored at −80°C until analyzed 14.

### Body composition and abdominal fat distribution

Dual energy X‐ray absorptiometry (DXA, Lunar DPX‐Lunar Radiation, Madison WI) assessed body composition. Computed tomography (CT; Philips Gemini GXL) assessed abdominal fat distribution at L2/L3 and L4/L5, and liver and spleen attenuation 14. The ratio between attenuation of liver and spleen was the indicator of liver fat.

For biochemical analysis in blood and Western blotting in muscle, refer to Supporting Information.

### Plasma and muscle lipidomics

Lipid species of the following classes were measured: sphingosine (SPH), dhCer, Cer, HexCer, Hex2Cer, Hex3Cer, GM3, SM, phosphatidylcholine (PC), alkylphosphatidylcholine (PC‐O), alkenylphosphatidylcholine (plasmalogen, PC‐P), lysophosphatidylcholine (LPC), lysoalkylphosphatidylcholine (LPC‐O), phosphatidylethanolamine (PE), alkylphosphatidylethanolamine (PE‐O), alkenylphosphatidylethanolamine (plasmalogen, PE‐P), lysophosphatidylethanolamine (LPE), phosphatidylinositol (PI), phosphatidylserine (PS), phosphatidylglycerol (PG), cholesterol ester (CE), free cholesterol (COH), DG, and TG. For detail, refer to Supporting Information.

### Gene array analysis

Two microarray‐based GE experiments were conducted on muscle obtained from the original cohort 14. The first experiment used Agilent chips 14. The second was performed in another set of individuals from that cohort. The overall GE analysis comprised a subset (Lean = 13, OIR = 11 and OIS = 11) which is the common subset of individuals where both transcriptomics and lipidomics data were available. GE profiles were obtained using both Affymetrix U133A and Agilent Whole Mouse Genome 4x44K array platforms. The relative mRNA levels for all transcripts across all subjects were studied. The raw Agilent data were pre‐processed 14. GE measures obtained for ∼56K transcripts using the Affymetrix U133A array platform were pre‐processed using Robust Multi‐array Average (RMA) method 15 in the R‐programming environment.

### Integrating GE data from two different microarray platforms and statistical analysis

To increase the power of data analysis, we integrated GE data obtained from Agilent and Affymetrix technologies using the Removal of Unwanted Variation (RUV‐4) method 16 within R‐package ruv (version 0.9.4). The probe sets in Agilent and Affymetrix arrays were collapsed to gene level by retaining the median expression of all probes for a particular gene. The common subset of highly expressed genes (log intensity>5) between the two array platforms was 17100. To remove platform dependent batch effects using RUV‐4, we curated a set of positive control genes (*n* = 280) from KEGG database 17 using genes from the insulin signaling, mTOR, type 2 diabetes, PPAR signaling and glycolysis pathways. Next, we obtained a predefined set of negative control or housekeeping genes 18 and removed overlapping positive control genes (*n* = 350). RUV‐4 requires selection of the number *K* of unwanted factors to adjust for. Using positive and negative control gene sets, we determined an appropriate choice of *K* to be *K* = 7 for comparing OIS versus Lean, *K* = 10 for OIR versus OIS, and *K* = 2 for OIR versus Lean. The differential expression analysis performed within RUV‐4 results in ranked lists of genes that were altered between the different groups compared (multiple hypothesis testing correction was performed and empirical Bayes 19 used for global variance shrinkage). Kolmogorov–Smirnov‐based gene set enrichment tests 20 determined which of the KEGG pathways were up‐ or downregulated in the GE data using *t*‐statistic of the genes as the test statistic. The KEGG pathways (*n* = 191) were obtained from the Molecular Signatures Database v4.0 (MSigDB)'s “C2 curated gene set” which is built from online pathway databases, publications in PubMed, and knowledge of domain experts. Significance was set on P<0.05.

### Statistical analysis of clinical, metabolic, and lipidomics data

Lipidomics data were log10‐transformed and differences between groups assessed using one‐way ANOVA and expressed as log‐fold change. Using the method outlined in a previous lipidomics report by our group 21, significance determination and control for multiple comparisons was made by assessing the distribution of the *P* values resulting for each lipid between cohorts. If the data were random then the relationship between the *P* values and their rank would be linear. For data in this study the relationships were clearly nonlinear for *P* ≤ 0.05 (not shown), therefore, *P* ≤ 0.05 was considered significant. Pearson's correlations were calculated between lipids common in plasma and muscle, or between lipids and clinical and metabolic factors and presented as heat maps with the color indicating the pairwise linear correlation coefficient for those pairs with *P* < 0.01. Statistical analyses of clinical and metabolic data were performed using SPSS (v22) and lipidomics using Matlab R2013b (Mathworks).

## Results

### Clinical and metabolic characteristics of subjects

OIS and OIR had similar BMI and body fat (Table 1). L2/L3 and L4/L5 visceral and subcutaneous adipose tissue areas were higher in both OIS and OIR versus Lean, and not different between OIS and OIR (*P* > 0.08). In the L4/L5 subcutaneous area, OIS had significantly greater superficial fat versus Lean and both OIS and OIR had more deep subcutaneous fat versus Lean. Liver fat, measured as CT attenuation of liver, was significantly higher in OIR versus Lean. OIS were not different from either Lean (*P* = 0.2) or OIR (*P* = 0.7). Systolic blood pressure was significantly higher in OIR compared with both OIS and Lean. Diastolic blood pressure was significantly higher in both obesity groups, and not different between OIS and OIR (*P* = 0.1). By design, insulin sensitivity measured by clamp was not different between Lean and OIS (*P* = 1), but was markedly reduced in OIR versus Lean and OIS (*P* ≤ 0.001). While baseline RQ was not different between groups, the change in RQ during hyperinsulinemia (ΔRQ), reflecting metabolic flexibility, was significantly lower in OIR versus both Lean and OIS. Fasting glucose and insulin were significantly greater in OIR versus Lean. HOMA‐IR was greater in both OIR (*P* < 0.001) and OIS (*P* = 0.02) versus Lean, and also greater in OIR versus OIS (*P* = 0.04). Fasting NEFA were not different between groups, adiponectin was significantly lower in OIR versus Lean. FABP4 was higher in both OIR and OIS versus Lean, while FGF‐21 was not different between groups.

**Table 1:** Clinical and metabolic characteristics of the cohort

		Overweight/obesity		
	Lean	Insulin‐sensitive (OIS)	Insulin‐resistant (OIR)	ANOVA *P* value
**N (M/F)**	23 (9/14)	14 (7/7)	14 (7/7)	
**Age (years)**	55 ± 2	56 ± 3	58 ± 2	0.6
**BMI (kg/m^2^)**	21.9 ± 0.4	31.0 ± 1.2^**^	33.1 ± 2.1^**^	<0.001
**Body fat (%)**	27 ± 2	40 ± 2^**^	42 ± 3^**^	<0.001
**L2/L3 visceral area (cm^2^)**	46 ± 6	190 ± 42^**^	231 ± 27^**^	<0.001
**L2/L3 subcutaneous area (cm^2^)**	86 ± 11	233 ± 28^**^	248 ± 41^**^	<0.001
**L4/L5 visceral area (cm^2^)**	56 ± 5	126 ± 24^**^	177 ± 18^**^	<0.001
**L4/L5 subcutaneous area (cm^2^)**	151 ± 15	356 ± 41^**^	382 ± 52^**^	<0.001
**L4/L5 superficial subcutaneous area (cm^2^)**	83 ± 10	153 ± 23^**^	113 ± 15	0.01
**L4/L5 deep subcutaneous area (cm^2^)**	83 ± 9	206 ± 33^**^	207 ± 32^**^	0.001
**CT attenuation liver/spleen ratio**	1.35 ± 0.07	1.11 ± 0.12	0.99 ± 0.13^*^	0.03
**Systolic blood pressure (mm Hg)**	120 ± 3	125 ± 4	140 ± 4^**#^	0.001
**Diastolic blood pressure (mm Hg)**	73 ± 2	81 ± 2^*^	87 ± 2^**^	<0.001
**Glucose infusion rate (µmol/min/kg FFM)**	92 ± 5	90 ± 10	54 ± 5^**##^	<0.001
**RQ_Baseline_**	0.81 ± 0.01	0.82 ± 0.01	0.82 ± 0.02	0.6
**Δ RQ (RQ_Baseline_‐RQ_Clamp_)**	0.13 ± 0.01	0.11 ± 0.01	0.06 ± 0.02^**#^	<0.001
**Fasting blood glucose (mmol/L)**	4.5 ± 0.1	4.9 ± 0.1	5.2 ± 0.2^**^	<0.001
**Fasting serum insulin (mU/L)**	12 ± 1	16 ± 2	22 ± 2^**#^	<0.001
**HOMA‐IR**	1.1 ± 0.1	2.3 ± 0.4^*^	3.6 ± 0.5^**#^	<0.001
**Fasting serum NEFA (mmol/L)**	0.37 ± 0.03	0.32 ± 0.04	0.35 ± 0.03	0.6
**Fasting serum adiponectin (mg/L)**	25 ± 3	20 ± 3	16 ± 2^*^	0.04
**Fasting serum FABP4 (µg/L)**	15 ± 2	30 ± 5^*^	29 ± 5^*^	0.01
**Fasting serum FGF‐21 (ng/L)**	93 ± 18	108 ± 24	159 ± 29	0.1
**pAkt474 baseline** a	1.0 ± 0.1	1.0 ± 0.2	1.2 ± 0.2	0.7
**pAkt474 30 min** a	6.4 ± 0.5	6.6 ± 0.9	6.5 ± 0.5	1.0
**pAkt474 145 min** a	7.8 ± 0.5	7.4 ± 0.8	7.4 ± 0.7	0.9
**pAkt309 baseline** a	1.0 ± 0.1	1.0 ± 0.1	1.3 ± 0.2	0.5
**pAkt309 30 min** a	9.3 ± 0.8	7.9 ± 1.1	7.0 ± 0.8	0.3
**pAkt309 145 min** a	12.1 ± 1.1	8.9 ± 1.1	5.7 ± 1.4^**^	0.002
**pAS160 baseline** a	1.0 ± 0.2	0.8 ± 0.1	0.8 ± 0.2	0.7
**pAS160 30 min** a	2.6 ± 0.4	1.7 ± 0.2	1.5 ± 0.2	0.07
**pAS160 145 min** a	2.7 ± 0.3	1.7 ± 0.2^*^	1.4 ± 0.6^*^	0.005

Data are mean ± SEM.

a Expression of skeletal muscle phosphorylated proteins relative to Lean average at baseline.

BMI, body mass index; CT, computed tomography; FABP, fatty acid binding protein; FFM, fat‐free mass; FGF, fibroblast growth factor; NEFA, nonesterified fatty acid; RQ, respiratory quotient.

Differences versus the Lean group ^*^ *P*<0.05 and ^**^ *P*<0.01 and versus the OIS group ^#^ *P*<0.05 and ^##^ *P*<0.01 by one‐way ANOVA and Tukey post hoc analyses.

Western blot of key insulin signaling intermediates in muscle were examined at baseline and during clamp hyperinsulinemia (Table 1). While pAkt474 expression was not different between groups at any time point (*P* ≥ 0.7), pAkt309 expression at 145 min was significantly lower in OIR versus Lean (*P* = 0.002). However, it was not different between OIR and OIS (*P* = 0.2) or between OIS and Lean (*P* = 0.2). pAS160 expression at 145 min was significantly lower in both OIR (*P* = 0.01) and OIS (*P* = 0.04) versus Lean, but was not different between OIR and OIS (*P* = 0.7). These findings are consistent with findings previously reported in the whole cohort 14.

### Lipids specific to insulin resistance

Full list of lipids detected and within class abundance in plasma and muscle are in Supporting Information Table 1.

**Figure 1:**
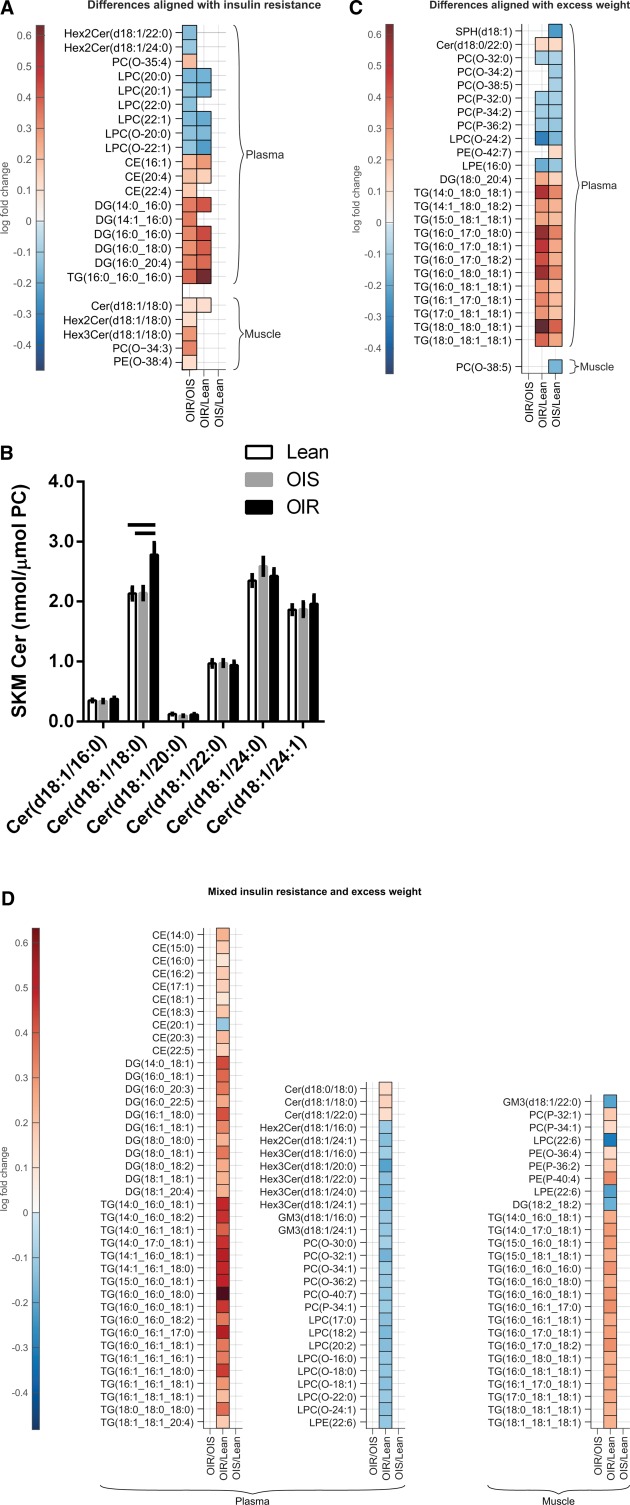
Differences in concentrations of lipid species in plasma and skeletal muscle, including differences that align with (**A**) insulin resistance, (**B**) skeletal muscle concentrations of ceramide species, (**C**) differences between lipids in plasma and skeletal muscle that align with overweight/obesity, and (**D**) mixed differences. The columns in panels A, C, and D represent changes between OIR versus OIS, OIR versus Lean, and OIS versus Lean, respectively, and only lipids with significant differences between the cohorts are shown. Values in panels A, C, and D are log‐fold changes of significant increases (red) and decreases (blue). Values in panel B are mean ± SEM, and statistical significance by one‐way ANOVA (*P* < 0.05) is depicted by bars across.

In plasma, insulin resistance was aligned with higher TG(16:0_16:0_16:0), DG(14:0_16:0), DG(16:0_16:0), DG(16:0_18:0), and DG(16:0_20:4), CE(16:1) and CE(20:4) and lower LPC(20:0), LPC(20:1), LPC(22:1), LPC(O‐20:0) and LPC(O‐22:1) (Figure 1A). Plasma lipids specific to obesity‐related insulin resistance (i.e., different between OIS and OIR, but not between OIR and Lean) included DG(14:1_16:0), CE(22:4) and PC(O‐35:4) (higher) and Hex2Cer(d18:1/22:0), Hex2Cer(d18:1/24:0) and LPC(22:0) (lower).

In muscle, Cer(d18:1/18:0) was the only lipid higher in insulin resistance independent of overweight/obesity. Other chain lengths Cer were not different between groups (Figure 1B). Other muscle lipid differences specific to insulin resistance in obesity included higher concentrations of C18:0 sphingolipids Hex2Cer and Hex3Cer, PC(O‐34:3) and PE(O‐38:4).

### Lipids specific to obesity

An overweight/obesity signature was only apparent in plasma (Figure 1C), including the higher dhCer Cer(d18:0/22:0), 12 TG species, including the most abundant TG(16:0_18:1_18:1) and DG(18:0_20:4). Lipids lower in overweight/obesity included PC(O), PC(P), LPC(O‐24:2), and LPE(16:0).

### Lipids with mixed contributions from insulin resistance and adiposity

In many lipids in plasma and muscle, a clear insulin resistance or overweight/obesity lipid signature was not apparent, but mixed effects related to insulin resistance and adiposity were observed (Figure 1D). In plasma, a large proportion of TG, DG, and CE were higher in insulin resistance. Cer(d18:1/18:0), its precursor Cer(d18:0/18:0), and Cer(d18:1/22:0) were also higher. Conversely, many phospholipids were lower in OIR versus Lean, including a large proportion of plasma LPC(O), LPC, and PC(O) species, and PC(P‐34:1). Sphingolipids that were lower in OIR versus Lean included the most and second most abundant Hex2Cer(d18:1/16:0) and Hex2Cer(d18:1/24:1), 5 of 6 Hex3Cer including the most abundant Hex3Cer(d18:1/16:0) and the two most abundant GM3 GM3(d18:1/16:0) and GM3(d18:1/24:1). In muscle, lipid species higher in OIR versus Lean included a large proportion of TG, PC(P‐32:1) and PC(P‐34:1), PE(O‐36:4), and PE(P‐36:2) and PE(P‐40:4). Lower muscle lipids in OIR versus Lean included GM3(d18:1/22:0), LPC(22:6), LPE(22:6), and DG(18:2_18:2).

### Relationships between glycerolipids, sphingolipids, and CE common in plasma and muscle

**Figure 2:**
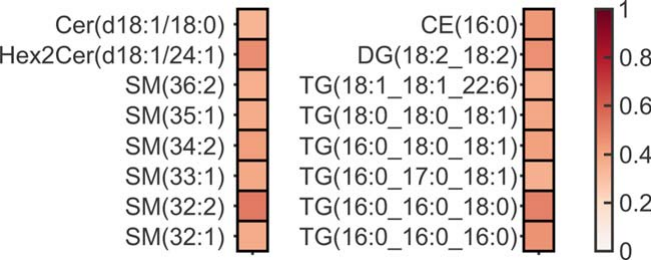
Heat map based on Pearson's correlations between fasting glycerolipids, sphingolipids, and cholesterol esters common in plasma and muscle. The *R* value of the correlation is shown in the color, all *P* < 0.01.

Of the sphingolipids detected in both plasma and muscle (*n* = 40), only Cer(d18:1/18:0), Hex2Cer(d18:1/24:1) and 6 SM species (of 17) correlated significantly (Figure 2). Plasma and muscle concentrations of DG(18:2_18:2) and 6 TG species also correlated.

### Relationships between muscle and plasma lipids and adiposity, metabolic factors, and blood pressure

Relationships between plasma and muscle lipids with clinical and metabolic factors were assessed. In plasma (Supporting Information, Figure 1A), almost all glycerolipids correlated positively with visceral fat and many correlated with liver fat. Of the circulating factors, insulin and FGF‐21 correlated positively and adiponectin inversely with many glycerolipids, but FABP4 did not correlate with any. Metabolic flexibility (ΔRQ) correlated inversely with the majority of glycerolipids. Systolic blood pressure correlated positively with selected TG and DG species, but diastolic blood pressure did not correlate with any plasma lipid. Of the sphingolipids, dhCer C18:0 and all saturated Cer correlated positively with visceral fat and some positively with FGF‐21 and inversely with adiponectin. Plasmalogens and LPC species correlated inversely with adiposity, liver fat and insulin. In muscle (Supporting Information, Figure 1B), the majority of TG correlated positively with subcutaneous fat and some correlated positively with insulin and inversely with ΔRQ. Of the sphingolipids, Cer C18:0 correlated positively with visceral and liver fat, and with systolic and diastolic blood pressure, and inversely with ΔRQ. Similar relationships were demonstrated between metabolic factors and muscle GM3 C18:0. Interestingly, muscle sphingolipids and glycerolipids did not correlate with the insulin signaling intermediates measured at any time point (not shown).

### Gene expression

**Figure 3:**
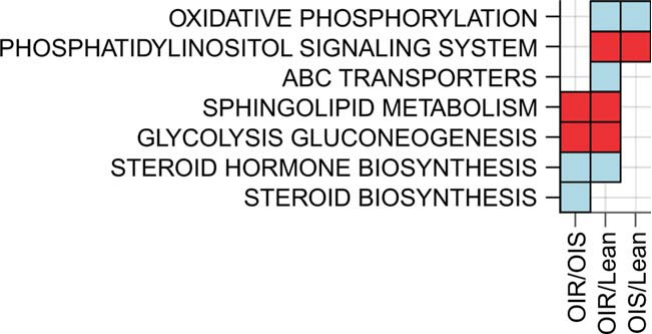
Lipid‐related pathways altered in gene expression data when comparing the three groups OIR, OIS, and Lean. The columns represent changes between OIR versus OIS, OIR versus Lean, and OIS versus Lean, respectively. Kolmogorov‐Smirnov‐based gene set enrichment tests were used to determine which of the KEGG pathways were up‐ (red) or downregulated (blue) in the GE data using t‐statistic of the genes as the test statistic from differential expression analysis between the different groups compared. Significance was defined by *P* value < 0.05. Figure generated using package ggplot 2 in *R*.

Lipid‐related pathway analysis of muscle gene expression was performed in a representative subset (for gene array subcohort characteristics see Supporting Information, Table 2; for a comprehensive list of pathways that were significantly up‐ or downregulated between groups, see Supporting Information, Table 3). The sphingolipid metabolism and glycolysis/gluconeogenesis pathways were upregulated in OIR versus both OIS and Lean (Figure 3), suggesting that upregulation of these pathways characterize insulin resistance irrespective of obesity. Similarly, the steroid hormone biosynthesis pathway was downregulated in insulin resistance irrespective of obesity. On the other hand, oxidative phosphorylation was downregulated in both OIS and OIR compared with Lean, suggesting that downregulation of oxidative phosphorylation characterizes obesity *per se*. Similarly, the phosphatidylinositol signaling system was upregulated in obesity irrespective of insulin resistance. The ABC transporters pathway was downregulated in OIR versus Lean only (Figure 3). Fifty seven and 32 (out of 17,100) genes were significantly up‐ or downregulated between OIR and Lean and OIR and OIS, respectively (corrected *P* < 0.05). Interestingly, no genes were differentially regulated between OIS and Lean (corrected *P* > 0.05). Within the sphingolipid pathway, ceramide synthase (CERS)3 and CERS6 were 32 and 19% upregulated in OIR versus OIS, respectively and 24 and 15% in OIR versus Lean, respectively. Galactosylceramidase (GALC) was upregulated in OIR versus Lean (20%). CERS1, the enzyme specific for C18:0 ceramide generation 22, was not mapped to the gene array.

## Discussion

Lipidomic analysis of plasma and skeletal muscle revealed signatures of insulin resistance and overweight/obesity. The latter was only apparent in plasma and dominated by increases in TG and decreases in plasmalogen species. Conversely, insulin resistance was characterized in plasma by higher DG and CE and lower LPC species, while in muscle insulin resistance was predominantly associated with higher C18:0 sphingolipids.

This study identifies the long‐chain C18:0 ceramide in muscle as a potential player in insulin resistance in humans. C18:0 ceramide is the most abundant ceramide in muscle in rodents 5, 22 and equally most abundant with C24:0 ceramide in muscle in this study (∼30% each). In mice, muscle C18:0 ceramide was the first and only ceramide to increase with 3‐weeks of high fat feeding and induction of peripheral insulin resistance 5. In humans, other cross‐sectional studies have reported increases in some 23, 24 or all 25 saturated ceramides in insulin‐resistant subjects with obesity. Coen and colleagues 8 have demonstrated higher concentrations of selected saturated ceramides, including C14:0, C16:0, and C18:0 in insulin‐resistant versus insulin‐sensitive postmenopausal women with obesity. In this study, ceramides other than C18:0 were not different between groups. In a small interventional study, Dube and colleagues reported decreases in different chain length ceramides with exercise and caloric restriction concomitant with improved insulin sensitivity, and while C18:0 ceramide decreased with exercise, its concentration was unchanged with caloric restriction 26. Similarly, decreases were noted in muscle C18:0 ceramide with bariatric surgery and exercise, but not with surgery alone, although both interventions improved insulin sensitivity; however, this may have been related to higher concentrations of C18:0 ceramide pre‐exercise 27. These interventional studies demonstrate the complexity of the interactions between ceramides and insulin resistance in humans. Similarly, the role that muscle C16:0 ceramide plays in insulin resistance in humans is also unclear and findings from cross‐sectional studies are conflicting. Some, similar to our findings, reported no difference between insulin‐resistant and insulin‐sensitive individuals 23, 24, while others reported higher concentrations in insulin resistance 8. In any case, direct comparison between muscle lipidomic studies is complicated by different cohorts and different methodology. In all studies, including ours, cohort size was relatively small, resulting in greater variability and further complicating comparison between findings. In mice, however, C16:0 ceramide may be important in adipose tissue 5 and liver 4, 5, 6 insulin resistance. The role that individual ceramides play in insulin resistance is undergoing further research in animal models and the mechanisms by which different ceramide chain lengths and saturation induce insulin resistance await further study.

Accumulation of ceramides in tissues is determined by complex biosynthetic and degradative pathways. Numerous stimuli implicated in insulin resistance induce sphingolipid metabolism and determine ceramide metabolic fate. An inflammatory environment has been linked to upregulation of ceramides through toll‐like receptor‐4 activation that increases transcription of enzymes involved in *de novo* ceramide synthesis, and pro‐inflammatory cytokines can activate sphingomyelinase 28. Conversely, the adiponectin receptor is reported to have ceramidase activity 29 and FGF‐21 is reported to stimulate adiponectin secretion while diminishing ceramide accumulation, thereby mediating insulin sensitizing effects in obese rodents 30. Here we report that the sphingolipid metabolism pathway was upregulated in muscle in insulin resistance independent of obesity. The most upregulated genes were genes encoding the *de novo* ceramide biosynthetic enzymes CERS3 and CERS6. While CERS3 may influence accumulation of the very long‐chain ceramide C26 22 not detected here, CERS6 generates C16 ceramides in liver and skeletal muscle 22, although we did not detect higher C16 ceramide in either plasma or muscle in insulin resistance. CERS1, the ceramide synthase with C18 fatty acid specificity in skeletal muscle 22, was not mapped. GALC, the gene encoding a lysosomal enzyme that hydrolyzes galactose ester bonds of higher order sphingolipids was upregulated in OIR and may explain increases in muscle ceramides.

While an adiponectin‐FGF‐21‐ceramide axis has been proposed in animal models, correlations between these adipokines/hepatokines and muscle Cer were not impressive. However, plasma C18:0 was the only ceramide correlating positively with circulating FGF‐21 and inversely with adiponectin, further supporting a potential deleterious role for this ceramide in insulin resistance. Other than generation of ceramide in tissues, several lines of evidence suggest the liver is a major source of ceramides circulating on LDL and taken up by skeletal muscle in a LDL receptor‐independent manner 31. Boon and colleagues have reported that LDL‐Cer is increased in type 2 diabetes and decreased with caloric restriction in women 31. In the same study, infusion of LDL‐Cer to mice reduced glucose disposal in muscle in parallel with a tendency to increase plasma membrane ceramide 31. Positive associations between circulating C16:0 and C18:0 ceramides and markers of muscle NF‐_K_B activation 32 further strengthen the circulating ceramides‐muscle insulin resistance notion. LDL‐Cer were not measured here, but uniquely and unlike other ceramides, C18:0 ceramide concentration in plasma and muscle correlated positively, possibly placing C18:0 ceramide at that circulation‐muscle crosslink. Interestingly, only a small proportion of glycerolipids, sphingolipids and CEs common to plasma and muscle were correlated and further investigation is required to clarify the metabolic implications of these findings.

Plasma LPCs were lower in insulin resistance, irrespective of obesity. LPCs are carried primarily on HDL and albumin 33 and other metabolomics/lipidomics screens support our conclusions that increased circulating LPCs are indicators of metabolic health in obesity 34, 35, 36. These studies dissected the LPC signature of metabolic health in obesity, such that LPC(18:2) predicted lower liver fat 36 and LPC(16:0) differentiated between insulin‐sensitive and insulin‐resistant individuals with fatty liver 35. There remains a need to clarify the predictive capacity and role of specific LPC species in metabolic health in obesity.

The lipid signature of obesity *per se* was exclusive to plasma and included lower plasmalogen and higher TG species. However, many other TG, and other plasma lipids, were only different when comparing the OIR and Lean groups. For example, many DG were increased in OIR versus Lean, consistent with higher liver DG in individuals with fatty liver 37. Unlike findings in plasma in OIR versus Lean, only DG(18:2_18:2) was significantly different between groups in muscle and, contrary to DG in plasma, was lower. Other studies evaluating whole muscle DG 8, 23, 38 did not find differences between insulin‐resistant and insulin‐sensitive individuals. Similarly, the majority of CEs were upregulated in OIR versus Lean and may reflect differences in LDL concentrations. However, the upregulation of some CEs in OIR versus both OIS and Lean suggests LDL lipid composition may differ between insulin‐resistant and insulin‐sensitive individuals. Correlation studies with adiposity and metabolic markers revealed significant associations between visceral and liver fat and circulating glycerolipids and CE. Interestingly with muscle lipids, subcutaneous fat was a better predictor of TGs than visceral fat and, as expected, decreased metabolic flexibility associated with increased muscle TGs.

Large cohort studies, including studies from our group, have highlighted the strong potential of plasma lipidomics in explaining metabolic disease when added to traditional disease markers, including waist circumference, fasting glucose, and insulin 12, 13, 39. In the present study we refine these findings and differentiate between plasma lipids that align with insulin resistance from those aligning with excess adiposity. However, our findings are also consistent with the strong link between insulin resistance and obesity, with many lipids in tissues demonstrating mixed effects. Strengths of the study are the comprehensive lipidomics analysis of muscle and plasma in groups of individuals with equal excess adiposity diverging in insulin sensitivity and the inclusion of a lean insulin‐sensitive reference group, a unique design enabling dissection of effects of insulin resistance from overweight/obesity. Limitations include the analysis of lipids in whole muscle, rather than in subcellular fractions, and the limited investigation of potential mediators of ceramide‐induced insulin resistance in muscle. Lastly, lipidomics in fractionated lipoproteins was not performed and the association of specific lipoprotein lipids with insulin resistance and overweight/obesity awaits further study.

Insulin‐sensitive or metabolically healthy individuals with obesity are suggested to be relatively protected from type 2 diabetes in longitudinal studies. In this study, we identified C18:0 sphingolipids in muscle as potential players in insulin resistance in obesity. Longitudinal studies are required to establish whether insulin sensitivity in obesity is sustained over time and whether it can be explained by unique tissue lipid signatures in humans.
