# Supplementary material for: Skeletal muscle and plasma lipidomic signatures of insulin resistance and overweight/obesity in humans
Source: Obesity (Silver Spring). 2016 Feb 24;24(4):908–16. doi: 10.1002/oby.21448 (PMC6585800; doi:10.1002/oby.21448)
Supplement: Supplementary file 1 [file OBY-24-908-s001.pdf]

Skeletal muscle and plasma lipidomic signatures of insulin resistance and overweight/obesity in humans
Authors: Tonks, Coster, Christopher, Chaudhuri, Xu, Gagnon-Bartsch, Chisholm, James, Meikle, Greenfield and Samocha-Bonet
Contact: peter.meikle@bakeridi.edu.au or d.samochabonet@garvan.org.au

Supplementary Material

Methods

Biochemical analysis

Blood glucose was assessed by the glucose oxidase method (YSI 2300, Life Sciences), serum insulin by radioimmunoassay (Merck Millipore, Billerica, MA, US) and non-esterified fatty acids (NEFA) by an enzymatic colorimetric assay (Wako, Osaka, Japan). Adiponectin, fatty acid binding protein (FABP)-4 and fibroblast growth factor (FGF)-21 were determined by enzyme-linked immune-absorbent assays established by Antibody and Immunoassay Services, the University of Hong Kong, as described (1).

Western blotting in muscle

Tissue processing has been described in detail previously (1). Expression of insulin signalling intermediates in muscle lysates was determined using polyclonal antibodies. pAS160 was detected by Supersignal West Pico chemiluminescent substrate (Millipore Corporation, Billerica, MA) and Akt, pAkt474, pAkt309 (Cell Signaling Technologies, Beverly, MA) and AS160 (obtained from Peter Shepherd, Symansis, Auckland, New Zealand) by infrared dye 700- or 800-conjugated secondary antibodies. Quantification of protein was performed using Odyssey infrared imaging system software v2.0 (LI-COR Biosciences, Lincoln, NE). 14-3-3β was used as loading control. Phosphorylated protein data are expressed relative to mean expression in Lean at baseline.

Authors: Tonks, Coster, Christopher, Chaudhuri, Xu, Gagnon-Bartsch, Chisholm, James, Meikle, Greenfield and Samocha-Bonet
Contact: peter.meikle@bakeridi.edu.au or d.samochabonet@garvan.org.au

Methods – Contd.

Plasma and muscle lipidomics

Muscle samples were homogenized in 10 volumes of ice-cold PBS (pH 7.5) for 10-15 sec at 30,000 rpm with a PT 3100 electric homogeniser (Polytron), sonicated on ice for 15 sec at amplitude 23° using a digital probe-sonifier (Branson), assayed for total protein content in duplicate using the bicinchoninic acid (BCA) protein assay (Pierce Biotechnology BCA™ Protein Assay Kit), and the muscle homogenate diluted to a final stock concentration of 5 mg protein/ml with ice-cold PBS (pH 7.5), and stored at -80°C. Lipids were extracted from plasma (10µL) and muscle homogenates (50µg protein) with a single phase chloroform/methanol (2:1 vol/vol;20 volumes) extraction method following the addition of a 10µL internal standard mixture (containing 20 internal standards). Lipid analysis was performed by liquid chromatography, electrospray ionization-tandem mass spectrometry using an Agilent 1200 liquid chromatography system combined with an Applied Biosystems API 4000 Q/TRAP mass spectrometer with a turbo-ionspray source (350°C) and Analyst 1.5 data system, as described (2). Liquid chromatography was performed on a Zorbax C18, 1.8µm, 50×2.1 mm column (Agilent Technologies). Solvents A and B consisted of tetrahydrofuran:methanol:water in the ratio (30:20:50) and (75:20:5) respectively, both containing 10mM ammonium formate. Columns were heated to 50°C and the auto-sampler regulated to 25°C. DG and TG species (1µL injection) were separated using an isocratic flow (100µL/min) of 85% B over 6 min. All other lipid species (5µL injection) were separated under gradient conditions (300µL/min) 0% B to 100% B over 8.0 min, 2.5 min at 100% B, a return to 0% B over 0.5 min, then 10.5 min at 0% B prior to the next injection. Allowing for 0.5 min injection time, this equated to 14 min between injections. Lipid species of the

Authors: Tonks, Coster, Christopher, Chaudhuri, Xu, Gagnon-Bartsch, Chisholm, James, Meikle, Greenfield and Samocha-Bonet
Contact: peter.meikle@bakeridi.edu.au or d.samochabonet@garvan.org.au

Methods – Contd.

following classes were measured: sphingosine (SPH), dhCer, Cer, HexCer, Hex2Cer, Hex3Cer, GM3, SM, phosphatidylcholine (PC), alkylphosphatidylcholine (PC-O), alkenylphosphatidylcholine (plasmalogen, PC-P), lysophosphatidylcholine (LPC), lysoalkylphosphatidylcholine (LPC-O), phosphatidylethanolamine (PE), alkylphosphatidylethanolamine (PE-O), alkenylphosphatidylethanolamine (plasmalogen, PE-P), lysophosphatidylethanolamine (LPE), phosphatidylinositol (PI), phosphatidylserine (PS), phosphatidylglycerol (PG), cholesterol ester (CE), free cholesterol (COH), DG and TG. For a number of the lipids which contain two fatty acid chains, the multiple reaction monitoring (MRM) based measurements do not directly determine the constituent fatty acids, but the sum of the number of carbons and double bonds across both fatty acids. Accordingly, we denote these species as the combined length and number of double bonds. A total of 71 and 59 DG and TG and 266 and 238 other lipid species in plasma and muscle respectively were analyzed. Lipid concentrations were calculated by relating the peak area of each species to the peak area of the corresponding stable isotope or non-physiological internal standard (2). The lipidomic analysis used in this study represents semi-quantitative measurements of over 300 lipid species. The lack of availability of suitable stable isotope internal standards for every individual species requires the use of representative standards for each lipid class and precludes the creation of calibration curves for each lipid species. Thus, care must be taken in the interpretation of the data. Whilst the comparison of lipid species between individuals will provide good estimates of differences in lipid abundance (i.e., high assay precision), exact quantification and subsequent distribution of lipids within a class should be recognized as approximations only.

Authors: Tonks, Coster, Christopher, Chaudhuri, Xu, Gagnon-Bartsch, Chisholm, James, Meikle, Greenfield and Samocha-Bonet
Contact: peter.meikle@bakeridi.edu.au or d.samochabonet@garvan.org.au

Methods – Contd.

1. Tonks KT, Ng Y, Miller S, Coster ACF, Samocha-Bonet D, Iseli TJ, *et al.* Impaired Akt phosphorylation in insulin-resistant human muscle is accompanied by selective and heterogeneous downstream defects. *Diabetologia* 2013;**56**: 875-885.

2. Weir JM, Wong G, Barlow CK, Greeve MA, Kowalczyk A, Almasy L, *et al.* Plasma lipid profiling in a large population-based cohort. *Journal of Lipid Research* 2013;**54**: 2898-2908.

Skeletal muscle and plasma lipidomic signatures of insulin resistance and overweight/obesity in humans

Authors: Tonks, Coster, Christopher, Chaudhuri, Xu, Gagnon-Bartsch, Chisholm, James, Meikle, Greenfield and Samocha-Bonet

Contact: peter.meikle@bakeridi.edu.au or d.samochabonet@garvan.org.au

Supplementary Table 1: Lipid species in plasma and skeletal muscle analyses. Classes are sorted alphabetically, most abundant within class marked in bold

Plasma:

Class	Component	Mean percent of class	SEM
Alkenylphosphatidylcholine (PC-P)	PC(P-32:0)	4.8	0.1
Alkenylphosphatidylcholine (PC-P)	PC(P-32:1)	0.9	0.0
Alkenylphosphatidylcholine (PC-P)	PC(P-34:1)	9.2	0.3
Alkenylphosphatidylcholine (PC-P)	PC(P-34:2)	19.9	0.4
Alkenylphosphatidylcholine (PC-P)	PC(P-34:3)	0.6	0.0
Alkenylphosphatidylcholine (PC-P)	PC(P-36:2)	7.9	0.2
**Alkenylphosphatidylcholine (PC-P)**	**PC(P-36:4)**	**24.3**	**0.6**
Alkenylphosphatidylcholine (PC-P)	PC(P-36:5)	3.7	0.3
Alkenylphosphatidylcholine (PC-P)	PC(P-38:5)	20.9	0.4
Alkenylphosphatidylcholine (PC-P)	PC(P-40:5)	7.8	0.2
Alkenylphosphatidylethanolamine (PE-P)	PE(P-34:1)	1.7	0.1
Alkenylphosphatidylethanolamine (PE-P)	PE(P-34:2)	2.6	0.1
Alkenylphosphatidylethanolamine (PE-P)	PE(P-36:1)	1.8	0.1
Alkenylphosphatidylethanolamine (PE-P)	PE(P-36:2)	6.4	0.3
Alkenylphosphatidylethanolamine (PE-P)	PE(P-36:4)	10.4	0.3
Alkenylphosphatidylethanolamine (PE-P)	PE(P-38:4)	20.3	0.5
Alkenylphosphatidylethanolamine (PE-P)	**PE(P-38:5)**	**27.8**	**0.5**
Alkenylphosphatidylethanolamine (PE-P)	PE(P-38:6)	8.0	0.2
Alkenylphosphatidylethanolamine (PE-P)	PE(P-40:4)	1.4	0.1
Alkenylphosphatidylethanolamine (PE-P)	PE(P-40:5)	12.1	0.3
Alkenylphosphatidylethanolamine (PE-P)	PE(P-40:6)	7.4	0.2
Alkylphosphatidylcholine (PC-O)	PC(O-30:0)	0.3	0.0
Alkylphosphatidylcholine (PC-O)	PC(O-32:0)	3.9	0.1
Alkylphosphatidylcholine (PC-O)	PC(O-32:1)	0.7	0.0
Alkylphosphatidylcholine (PC-O)	PC(O-32:2)	0.1	0.0
Alkylphosphatidylcholine (PC-O)	PC(O-34:0)	0.8	0.0
Alkylphosphatidylcholine (PC-O)	PC(O-34:1)	8.0	0.2
Alkylphosphatidylcholine (PC-O)	PC(O-34:2)	6.0	0.2
Alkylphosphatidylcholine (PC-O)	PC(O-34:4)	0.2	0.0
Alkylphosphatidylcholine (PC-O)	PC(O-35:4)	0.4	0.0
Alkylphosphatidylcholine (PC-O)	PC(O-36:0)	0.2	0.0
Alkylphosphatidylcholine (PC-O)	PC(O-36:1)	1.2	0.1
Alkylphosphatidylcholine (PC-O)	PC(O-36:2)	5.6	0.2
Alkylphosphatidylcholine (PC-O)	PC(O-36:3)	6.9	0.1
**Alkylphosphatidylcholine (PC-O)**	**PC(O-36:4)**	**20.8**	**0.3**
Alkylphosphatidylcholine (PC-O)	PC(O-36:5)	1.7	0.2
Alkylphosphatidylcholine (PC-O)	PC(O-38:4)	18.7	0.5

Authors: Tonks, Coster, Christopher, Chaudhuri, Xu, Gagnon-Bartsch, Chisholm, James, Meikle, Greenfield and Samocha-Bonet
Contact: peter.meikle@bakeridi.edu.au or d.samochabonet@garvan.org.au

Supplementary Table 1-Contd.

Class	Component	Mean percent of class	SEM
Alkylphosphatidylcholine (PC-O)	PC(O-38:5)	20.6	0.2
Alkylphosphatidylcholine (PC-O)	PC(O-40:7)	3.9	0.2
Alkylphosphatidylethanolamine (PE-O)	PE(O-34:1)	2.5	0.1
Alkylphosphatidylethanolamine (PE-O)	PE(O-34:2)	1.7	0.1
Alkylphosphatidylethanolamine (PE-O)	PE(O-36:2)	3.4	0.1
Alkylphosphatidylethanolamine (PE-O)	PE(O-36:3)	2.9	0.1
Alkylphosphatidylethanolamine (PE-O)	PE(O-36:4)	10.1	0.3
Alkylphosphatidylethanolamine (PE-O)	PE(O-36:5)	1.9	0.1
Alkylphosphatidylethanolamine (PE-O)	PE(O-36:6)	1.5	0.2
Alkylphosphatidylethanolamine (PE-O)	PE(O-38:4)	12.9	0.4
Alkylphosphatidylethanolamine (PE-O)	PE(O-38:5)	14.7	0.3
**Alkylphosphatidylethanolamine (PE-O)**	**PE(O-38:6)**	**37.1**	**0.7**
Alkylphosphatidylethanolamine (PE-O)	PE(O-40:5)	4.3	0.2
Alkylphosphatidylethanolamine (PE-O)	PE(O-40:6)	2.1	0.1
Alkylphosphatidylethanolamine (PE-O)	PE(O-40:7)	4.5	0.2
Alkylphosphatidylethanolamine (PE-O)	PE(O-42:7)	0.3	0.0
Ceramide (Cer)	Cer(d18:1/16:0)	6.5	0.1
Ceramide (Cer)	Cer(d18:1/18:0)	2.6	0.1
Ceramide (Cer)	Cer(d18:1/20:0)	2.6	0.1
Ceramide (Cer)	Cer(d18:1/22:0)	16.0	0.2
**Ceramide (Cer)**	**Cer(d18:1/24:0)**	**52.7**	**0.6**
Ceramide (Cer)	Cer(d18:1/24:1)	19.7	0.4
Cholesteryl ester (CE)	CE(14:0)	0.7	0.0
Cholesteryl ester (CE)	CE(15:0)	0.6	0.0
Cholesteryl ester (CE)	CE(16:0)	12.9	0.3
Cholesteryl ester (CE)	CE(16:1)	5.8	0.3
Cholesteryl ester (CE)	CE(16:2)	0.2	0.0
Cholesteryl ester (CE)	CE(17:0)	0.3	0.0
Cholesteryl ester (CE)	CE(17:1)	0.4	0.0
Cholesteryl ester (CE)	CE(18:0)	0.5	0.0
Cholesteryl ester (CE)	CE(18:1)	14.7	0.3
**Cholesteryl ester (CE)**	**CE(18:2)**	**37.4**	**0.6**
Cholesteryl ester (CE)	CE(18:3)	4.6	0.2
Cholesteryl ester (CE)	CE(20:1)	0.0	0.0
Cholesteryl ester (CE)	CE(20:3)	0.9	0.0
Cholesteryl ester (CE)	CE(20:4)	13.5	0.4
Cholesteryl ester (CE)	CE(20:5)	5.2	0.4
Cholesteryl ester (CE)	CE(22:0)	0.0	0.0
Cholesteryl ester (CE)	CE(22:1)	0.0	0.0
Cholesteryl ester (CE)	CE(22:4)	0.0	0.0

Supplementary Table 1-Contd.

Class	Component	Mean percent of class	SEM
Cholesteryl ester (CE)	CE(22:5)	0.2	0.0
Cholesteryl ester (CE)	CE(22:6)	2.1	0.1
Cholesteryl ester (CE)	CE(24:0)	0.0	0.0
Cholesteryl ester (CE)	CE(24:1)	0.0	0.0
Cholesteryl ester (CE)	CE(24:4)	0.0	0.0
Cholesteryl ester (CE)	CE(24:5)	0.0	0.0
Cholesteryl ester (CE)	CE(24:6)	0.0	0.0
Diacylglycerol (DG)	DG(14:0_14:0)	0.1	0.0
Diacylglycerol (DG)	DG(14:0_16:0)	0.6	0.0
Diacylglycerol (DG)	DG(14:0_18:1)	1.8	0.1
Diacylglycerol (DG)	DG(14:0_18:2)	0.7	0.0
Diacylglycerol (DG)	DG(14:1_16:0)	0.1	0.0
Diacylglycerol (DG)	DG(16:0_16:0)	2.0	0.1
Diacylglycerol (DG)	DG(16:0_18:0)	1.5	0.1
Diacylglycerol (DG)	DG(16:0_18:1)	13.2	0.4
Diacylglycerol (DG)	DG(16:0_18:2)	5.7	0.2
Diacylglycerol (DG)	DG(16:0_20:0)	0.4	0.0
Diacylglycerol (DG)	DG(16:0_20:3)	0.4	0.0
Diacylglycerol (DG)	DG(16:0_20:4)	0.9	0.1
Diacylglycerol (DG)	DG(16:0_22:5)	0.4	0.0
Diacylglycerol (DG)	DG(16:0_22:6)	0.6	0.1
Diacylglycerol (DG)	DG(16:1_18:0)	0.5	0.0
Diacylglycerol (DG)	DG(16:1_18:1)	7.5	0.3
Diacylglycerol (DG)	DG(18:0_18:0)	0.5	0.1
Diacylglycerol (DG)	DG(18:0_18:1)	4.2	0.1
Diacylglycerol (DG)	DG(18:0_18:2)	1.5	0.0
Diacylglycerol (DG)	DG(18:0_20:4)	0.4	0.0
**Diacylglycerol (DG)**	**DG(18:1_18:1)**	**24.2**	**0.5**
Diacylglycerol (DG)	DG(18:1_18:2)	20.3	0.6
Diacylglycerol (DG)	DG(18:1_18:3)	3.3	0.1
Diacylglycerol (DG)	DG(18:1_20:0)	0.6	0.1
Diacylglycerol (DG)	DG(18:1_20:3)	1.7	0.1
Diacylglycerol (DG)	DG(18:1_20:4)	3.6	0.2
Diacylglycerol (DG)	DG(18:2_18:2)	3.3	0.2
**Dihexosylceramide (Hex2Cer)**	**Hex2Cer(d18:1/16:0)**	**69.5**	**0.6**
Dihexosylceramide (Hex2Cer)	Hex2Cer(d18:1/18:0)	1.2	0.0
Dihexosylceramide (Hex2Cer)	Hex2Cer(d18:1/20:0)	1.1	0.0
Dihexosylceramide (Hex2Cer)	Hex2Cer(d18:1/22:0)	6.2	0.3
Dihexosylceramide (Hex2Cer)	Hex2Cer(d18:1/24:0)	5.6	0.2
Dihexosylceramide (Hex2Cer)	Hex2Cer(d18:1/24:1)	16.4	0.3

Authors: Tonks, Coster, Christopher, Chaudhuri, Xu, Gagnon-Bartsch, Chisholm, James, Meikle, Greenfield and Samocha-Bonet
Contact: peter.meikle@bakeridi.edu.au or d.samochabonet@garvan.org.au

Supplementary Table 1-Contd.

Class	Component	Mean percent of class	SEM
Dihydroceramide (dhCer)	Cer(d18:0/16:0)	9.2	0.3
Dihydroceramide (dhCer)	Cer(d18:0/18:0)	11.9	0.3
Dihydroceramide (dhCer)	Cer(d18:0/20:0)	6.0	0.2
Dihydroceramide (dhCer)	Cer(d18:0/22:0)	20.3	0.4
**Dihydroceramide (dhCer)**	**Cer(d18:0/24:0)**	**33.4**	**0.6**
Dihydroceramide (dhCer)	Cer(d18:0/24:1)	19.3	0.4
**Free cholesterol (COH)**	**COH**	**100.0**	**0.0**
**G_{M3} ganglioside (GM3)**	**GM3(d18:1/16:0)**	**23.7**	**0.4**
G_{M3} ganglioside (GM3)	GM3(d18:1/18:0)	11.2	0.3
G_{M3} ganglioside (GM3)	GM3(d18:1/20:0)	6.7	0.1
G_{M3} ganglioside (GM3)	GM3(d18:1/22:0)	17.2	0.4
G_{M3} ganglioside (GM3)	GM3(d18:1/24:0)	17.5	0.4
G_{M3} ganglioside (GM3)	GM3(d18:1/24:1)	23.7	0.5
**Lysoalkylphosphatidylcholine (LPC-O)**	**LPC(O-16:0)**	**33.7**	**0.5**
Lysoalkylphosphatidylcholine (LPC-O)	LPC(O-18:0)	9.5	0.2
Lysoalkylphosphatidylcholine (LPC-O)	LPC(O-18:1)	21.2	0.5
Lysoalkylphosphatidylcholine (LPC-O)	LPC(O-20:0)	2.4	0.1
Lysoalkylphosphatidylcholine (LPC-O)	LPC(O-22:0)	4.8	0.1
Lysoalkylphosphatidylcholine (LPC-O)	LPC(O-22:1)	3.3	0.1
Lysoalkylphosphatidylcholine (LPC-O)	LPC(O-24:0)	11.9	0.4
Lysoalkylphosphatidylcholine (LPC-O)	LPC(O-24:1)	11.4	0.4
Lysoalkylphosphatidylcholine (LPC-O)	LPC(O-24:2)	1.7	0.1
Lysophosphatidylcholine (LPC)	LPC(14:0)	0.7	0.0
Lysophosphatidylcholine (LPC)	LPC(15:0)	0.5	0.0
**Lysophosphatidylcholine (LPC)**	**LPC(16:0)**	**47.5**	**0.5**
Lysophosphatidylcholine (LPC)	LPC(16:1)	1.6	0.1
Lysophosphatidylcholine (LPC)	LPC(17:0)	1.0	0.0
Lysophosphatidylcholine (LPC)	LPC(17:1)	0.2	0.0
Lysophosphatidylcholine (LPC)	LPC(18:0)	11.7	0.2
Lysophosphatidylcholine (LPC)	LPC(18:1)	12.0	0.2
Lysophosphatidylcholine (LPC)	LPC(18:2)	17.3	0.4
Lysophosphatidylcholine (LPC)	LPC(18:3)	0.4	0.0
Lysophosphatidylcholine (LPC)	LPC(20:0)	0.1	0.0
Lysophosphatidylcholine (LPC)	LPC(20:1)	0.1	0.0
Lysophosphatidylcholine (LPC)	LPC(20:2)	0.2	0.0
Lysophosphatidylcholine (LPC)	LPC(20:4)	3.9	0.1
Lysophosphatidylcholine (LPC)	LPC(20:5)	0.8	0.1
Lysophosphatidylcholine (LPC)	LPC(22:0)	0.0	0.0

Supplementary Table 1-Contd.

Class	Component	Mean percent of class	SEM
Lysophosphatidylcholine (LPC)	LPC(22:1)	0.0	0.0
Lysophosphatidylcholine (LPC)	LPC(22:5)	0.5	0.0
Lysophosphatidylcholine (LPC)	LPC(22:6)	1.5	0.1
Lysophosphatidylcholine (LPC)	LPC(24:0)	0.1	0.0
Lysophosphatidylcholine (LPC)	LPC(26:0)	0.0	0.0
Lysophosphatidylethanolamine (LPE)	LPE(16:0)	14.2	0.4
Lysophosphatidylethanolamine (LPE)	LPE(18:0)	17.3	0.4
Lysophosphatidylethanolamine (LPE)	LPE(18:1)	17.0	0.4
**Lysophosphatidylethanolamine (LPE)**	**LPE(18:2)**	**25.0**	**0.7**
Lysophosphatidylethanolamine (LPE)	LPE(20:4)	13.1	0.4
Lysophosphatidylethanolamine (LPE)	LPE(22:5)	2.3	0.1
Lysophosphatidylethanolamine (LPE)	LPE(22:6)	11.1	0.5
Monohexocylceramide (HexCer)	HexCer(d18:1/16:0)	10.6	0.2
Monohexocylceramide (HexCer)	HexCer(d18:1/18:0)	1.8	0.1
Monohexocylceramide (HexCer)	HexCer(d18:1/20:0)	2.6	0.1
Monohexocylceramide (HexCer)	HexCer(d18:1/22:0)	23.1	0.3
**Monohexocylceramide (HexCer)**	**HexCer(d18:1/24:0)**	**36.4**	**0.4**
Monohexocylceramide (HexCer)	HexCer(d18:1/24:1)	25.6	0.5
Phosphatidylcholine (PC)	PC(28:0)	0.0	0.0
Phosphatidylcholine (PC)	PC(29:0)	0.0	0.0
Phosphatidylcholine (PC)	PC(30:0)	0.3	0.0
Phosphatidylcholine (PC)	PC(31:0)	0.1	0.0
Phosphatidylcholine (PC)	PC(31:1)	0.1	0.0
Phosphatidylcholine (PC)	PC(32:0)	0.8	0.0
Phosphatidylcholine (PC)	PC(32:1)	1.8	0.1
Phosphatidylcholine (PC)	PC(32:2)	0.5	0.0
Phosphatidylcholine (PC)	PC(32:3)	0.0	0.0
Phosphatidylcholine (PC)	PC(33:0)	0.1	0.0
Phosphatidylcholine (PC)	PC(33:1)	0.3	0.0
Phosphatidylcholine (PC)	PC(33:2)	0.3	0.0
Phosphatidylcholine (PC)	PC(33:3)	0.0	0.0
Phosphatidylcholine (PC)	PC(34:0)	0.2	0.0
Phosphatidylcholine (PC)	PC(34:1)	9.9	0.1
**Phosphatidylcholine (PC)**	**PC(34:2)**	**17.9**	**0.3**
Phosphatidylcholine (PC)	PC(34:3)	1.4	0.0
Phosphatidylcholine (PC)	PC(34:4)	0.1	0.0
Phosphatidylcholine (PC)	PC(34:5)	0.0	0.0
Phosphatidylcholine (PC)	PC(35:0)	0.0	0.0
Phosphatidylcholine (PC)	PC(35:1)	0.0	0.0
Phosphatidylcholine (PC)	PC(35:2)	0.8	0.0

Supplementary Table 1-Contd.

Class	Component	Mean percent of class	SEM
Phosphatidylcholine (PC)	PC(35:3)	0.1	0.0
Phosphatidylcholine (PC)	PC(35:4)	0.0	0.0
Phosphatidylcholine (PC)	PC(35:5)	0.0	0.0
Phosphatidylcholine (PC)	PC(36:1)	4.2	0.1
Phosphatidylcholine (PC)	PC(36:2)	15.2	0.2
Phosphatidylcholine (PC)	PC(36:3)	8.1	0.1
Phosphatidylcholine (PC)	PC(36:4)	1.5	0.1
Phosphatidylcholine (PC)	PC(36:4)b	6.6	0.2
Phosphatidylcholine (PC)	PC(36:5)	2.6	0.2
Phosphatidylcholine (PC)	PC(36:6)	0.1	0.0
Phosphatidylcholine (PC)	PC(37:4)	0.0	0.0
Phosphatidylcholine (PC)	PC(37:5)	0.0	0.0
Phosphatidylcholine (PC)	PC(37:6)	0.1	0.0
Phosphatidylcholine (PC)	PC(38:2)	2.6	0.0
Phosphatidylcholine (PC)	PC(38:3)	4.9	0.1
Phosphatidylcholine (PC)	PC(38:4)	7.0	0.2
Phosphatidylcholine (PC)	PC(38:5)	4.4	0.1
Phosphatidylcholine (PC)	PC(38:6)a	0.6	0.0
Phosphatidylcholine (PC)	PC(38:6)b	3.6	0.1
Phosphatidylcholine (PC)	PC(38:7)	0.2	0.0
Phosphatidylcholine (PC)	PC(39:6)	0.1	0.0
Phosphatidylcholine (PC)	PC(40:5)	1.1	0.0
Phosphatidylcholine (PC)	PC(40:6)	1.7	0.1
Phosphatidylcholine (PC)	PC(40:7)	0.4	0.0
Phosphatidylethanolamine (PE)	PE(32:0)	0.4	0.0
Phosphatidylethanolamine (PE)	PE(32:1)	0.3	0.0
Phosphatidylethanolamine (PE)	PE(34:1)	3.9	0.1
Phosphatidylethanolamine (PE)	PE(34:2)	5.8	0.2
Phosphatidylethanolamine (PE)	PE(34:3)	0.4	0.0
Phosphatidylethanolamine (PE)	PE(35:1)	0.5	0.0
Phosphatidylethanolamine (PE)	PE(35:2)	0.4	0.0
Phosphatidylethanolamine (PE)	PE(36:0)	0.1	0.0
Phosphatidylethanolamine (PE)	PE(36:1)	3.2	0.1
Phosphatidylethanolamine (PE)	PE(36:2)	15.7	0.4
Phosphatidylethanolamine (PE)	PE(36:3)	5.4	0.2
Phosphatidylethanolamine (PE)	PE(36:4)	8.4	0.2
Phosphatidylethanolamine (PE)	PE(36:5)	0.8	0.1
Phosphatidylethanolamine (PE)	PE(38:3)	4.8	0.1
**Phosphatidylethanolamine (PE)**	**PE(38:4)**	**21.0**	**0.5**
Phosphatidylethanolamine (PE)	PE(38:5)	8.1	0.2

Authors: Tonks, Coster, Christopher, Chaudhuri, Xu, Gagnon-Bartsch, Chisholm, James, Meikle, Greenfield and Samocha-Bonet
Contact: peter.meikle@bakeridi.edu.au or d.samochabonet@garvan.org.au

Supplementary Table 1-Contd.

Class	Component	Mean percent of class	SEM
Phosphatidylethanolamine (PE)	PE(38:6)	10.5	0.4
Phosphatidylethanolamine (PE)	PE(40:5)	2.7	0.1
Phosphatidylethanolamine (PE)	PE(40:6)	6.5	0.3
Phosphatidylethanolamine (PE)	PE(40:7)	1.2	0.0
**Phosphatidylglycerol (PG)**	**PG(34:1)**	**52.3**	**1.3**
Phosphatidylglycerol (PG)	PG(36:1)	3.8	0.5
Phosphatidylglycerol (PG)	PG(36:2)	43.9	1.3
Phosphatidylinositol (PI)	PI(32:0)	0.5	0.0
Phosphatidylinositol (PI)	PI(32:1)	1.3	0.1
Phosphatidylinositol (PI)	PI(34:0)	0.2	0.0
Phosphatidylinositol (PI)	PI(34:1)	7.3	0.3
Phosphatidylinositol (PI)	PI(36:0)	0.0	0.0
Phosphatidylinositol (PI)	PI(36:1)	5.6	0.2
Phosphatidylinositol (PI)	PI(36:2)	16.8	0.5
Phosphatidylinositol (PI)	PI(36:3)	4.5	0.1
Phosphatidylinositol (PI)	PI(36:4)	5.4	0.2
Phosphatidylinositol (PI)	PI(38:2)	0.5	0.0
Phosphatidylinositol (PI)	PI(38:3)	11.3	0.2
**Phosphatidylinositol (PI)**	**PI(38:4)**	**37.8**	**0.7**
Phosphatidylinositol (PI)	PI(38:5)	3.5	0.1
Phosphatidylinositol (PI)	PI(38:6)	1.0	0.0
Phosphatidylinositol (PI)	PI(40:4)	0.5	0.0
Phosphatidylinositol (PI)	PI(40:5)	1.9	0.1
Phosphatidylinositol (PI)	PI(40:6)	2.1	0.1
Phosphatidylserine (PS)	PS(36:1)	35.0	1.0
Phosphatidylserine (PS)	PS(36:2)	6.0	0.2
Phosphatidylserine (PS)	PS(38:3)	9.7	0.2
**Phosphatidylserine (PS)**	**PS(38:4)**	**37.6**	**0.6**
Phosphatidylserine (PS)	PS(38:5)	3.3	0.1
Phosphatidylserine (PS)	PS(40:5)	4.1	0.3
Phosphatidylserine (PS)	PS(40:6)	4.3	0.3
Sphingomyelin (SM)	SM(31:1)	0.1	0.0
Sphingomyelin (SM)	SM(32:1)	3.8	0.1
Sphingomyelin (SM)	SM(32:2)	0.3	0.0
Sphingomyelin (SM)	SM(33:1)	2.2	0.0
**Sphingomyelin (SM)**	**SM(34:1)**	**39.8**	**0.4**
Sphingomyelin (SM)	SM(34:2)	5.8	0.1
Sphingomyelin (SM)	SM(34:3)	0.0	0.0
Sphingomyelin (SM)	SM(35:1)	1.5	0.0
Sphingomyelin (SM)	SM(35:2)	0.3	0.0

Supplementary Table 1-Contd.

Class	Component	Mean percent of class	SEM
Sphingomyelin (SM)	SM(36:1)	7.9	0.2
Sphingomyelin (SM)	SM(36:2)	3.9	0.1
Sphingomyelin (SM)	SM(36:3)	0.3	0.0
Sphingomyelin (SM)	SM(37:2)	0.1	0.0
Sphingomyelin (SM)	SM(38:1)	8.1	0.2
Sphingomyelin (SM)	SM(38:2)	2.4	0.1
Sphingomyelin (SM)	SM(39:1)	2.6	0.1
Sphingomyelin (SM)	SM(41:1)	6.9	0.1
Sphingomyelin (SM)	SM(41:2)	5.8	0.1
Sphingomyelin (SM)	SM(42:1)	8.1	0.2
**Sphingosine (SPH)**	**SPH(d18:1)**	**100.0**	**0.0**
Triacylglycerol (TG)	TG(14:0_16:0_18:1)	2.8	0.2
Triacylglycerol (TG)	TG(14:0_16:0_18:2)	1.7	0.1
Triacylglycerol (TG)	TG(14:0_16:1_18:1)	2.2	0.1
Triacylglycerol (TG)	TG(14:0_16:1_18:2)	0.4	0.0
Triacylglycerol (TG)	TG(14:0_17:0_18:1)	0.8	0.0
Triacylglycerol (TG)	TG(14:0_18:0_18:1)	0.2	0.0
Triacylglycerol (TG)	TG(14:0_18:2_18:2)	0.3	0.0
Triacylglycerol (TG)	TG(14:1_16:0_18:1)	0.5	0.0
Triacylglycerol (TG)	TG(14:1_16:1_18:0)	1.9	0.1
Triacylglycerol (TG)	TG(14:1_18:0_18:2)	0.1	0.0
Triacylglycerol (TG)	TG(14:1_18:1_18:1)	1.3	0.0
Triacylglycerol (TG)	TG(15:0_16:0_18:1)	0.4	0.0
Triacylglycerol (TG)	TG(15:0_18:1_18:1)	0.3	0.0
Triacylglycerol (TG)	TG(16:0_16:0_16:0)	0.8	0.1
Triacylglycerol (TG)	TG(16:0_16:0_18:0)	0.7	0.1
Triacylglycerol (TG)	TG(16:0_16:0_18:1)	9.8	0.3
Triacylglycerol (TG)	TG(16:0_16:0_18:2)	2.4	0.1
Triacylglycerol (TG)	TG(16:0_16:1_17:0)	0.9	0.1
Triacylglycerol (TG)	TG(16:0_16:1_18:1)	10.8	0.2
Triacylglycerol (TG)	TG(16:0_17:0_18:0)	0.0	0.0
Triacylglycerol (TG)	TG(16:0_17:0_18:1)	0.6	0.0
Triacylglycerol (TG)	TG(16:0_17:0_18:2)	0.9	0.0
Triacylglycerol (TG)	TG(16:0_18:0_18:1)	2.3	0.1
**Triacylglycerol (TG)**	**TG(16:0_18:1_18:1)**	**24.3**	**0.4**
Triacylglycerol (TG)	TG(16:0_18:1_18:2)	11.1	0.4
Triacylglycerol (TG)	TG(16:0_18:2_18:2)	2.9	0.2
Triacylglycerol (TG)	TG(16:1_16:1_16:1)	0.2	0.0
Triacylglycerol (TG)	TG(16:1_16:1_18:0)	0.2	0.0
Triacylglycerol (TG)	TG(16:1_16:1_18:1)	1.4	0.1

Supplementary Table 1-Contd.

Class	Component	Mean percent of class	SEM
Triacylglycerol (TG)	TG(16:1_17:0_18:1)	1.6	0.0
Triacylglycerol (TG)	TG(16:1_18:1_18:1)	2.3	0.1
Triacylglycerol (TG)	TG(16:1_18:1_18:2)	2.9	0.1
Triacylglycerol (TG)	TG(17:0_18:1_18:1)	0.7	0.0
Triacylglycerol (TG)	TG(18:0_18:0_18:0)	0.0	0.0
Triacylglycerol (TG)	TG(18:0_18:0_18:1)	0.2	0.0
Triacylglycerol (TG)	TG(18:0_18:1_18:1)	1.9	0.1
Triacylglycerol (TG)	TG(18:0_18:2_18:2)	0.4	0.0
Triacylglycerol (TG)	TG(18:1_18:1_18:1)	4.1	0.2
Triacylglycerol (TG)	TG(18:1_18:1_18:2)	0.9	0.1
Triacylglycerol (TG)	TG(18:1_18:1_20:4)	0.4	0.0
Triacylglycerol (TG)	TG(18:1_18:1_22:6)	0.4	0.0
Triacylglycerol (TG)	TG(18:1_18:2_18:2)	1.7	0.1
Triacylglycerol (TG)	TG(18:2_18:2_18:2)	0.2	0.0
Triacylglycerol (TG)	TG(18:2_18:2_20:4)	0.0	0.0
**Trihexosylceramide (Hex3Cer)**	**Hex3Cer(d18:1/16:0)**	**52.2**	**0.6**
Trihexosylceramide (Hex3Cer)	Hex3Cer(d18:1/18:0)	6.8	0.2
Trihexosylceramide (Hex3Cer)	Hex3Cer(d18:1/20:0)	2.5	0.1
Trihexosylceramide (Hex3Cer)	Hex3Cer(d18:1/22:0)	9.4	0.3
Trihexosylceramide (Hex3Cer)	Hex3Cer(d18:1/24:0)	11.2	0.3
Trihexosylceramide (Hex3Cer)	Hex3Cer(d18:1/24:1)	17.9	0.4

Supplementary Table 1-Contd.

Muscle:

Class	Component	Mean percent of class	SEM
Alkenylphosphatidylcholine (PC-P)	PC(P-32:0)	1.0	0.1
Alkenylphosphatidylcholine (PC-P)	PC(P-32:1)	0.7	0.0
Alkenylphosphatidylcholine (PC-P)	PC(P-34:1)	6.9	0.3
**Alkenylphosphatidylcholine (PC-P)**	**PC(P-34:2)**	**46.6**	**1.2**
Alkenylphosphatidylcholine (PC-P)	PC(P-34:3)	0.9	0.0
Alkenylphosphatidylcholine (PC-P)	PC(P-36:2)	8.2	0.3
Alkenylphosphatidylcholine (PC-P)	PC(P-36:4)	16.2	0.8
Alkenylphosphatidylcholine (PC-P)	PC(P-36:5)	4.1	0.5
Alkenylphosphatidylcholine (PC-P)	PC(P-38:4)	1.9	0.1
Alkenylphosphatidylcholine (PC-P)	PC(P-38:5)	9.4	0.4
Alkenylphosphatidylcholine (PC-P)	PC(P-38:6)	3.3	0.3
Alkenylphosphatidylcholine (PC-P)	PC(P-40:5)	0.1	0.0
Alkenylphosphatidylcholine (PC-P)	PC(P-40:6)	0.8	0.1
Alkenylphosphatidylethanolamine (PE-P)	PE(P-34:1)	2.2	0.1
Alkenylphosphatidylethanolamine (PE-P)	PE(P-34:2)	12.8	0.9
Alkenylphosphatidylethanolamine (PE-P)	PE(P-36:1)	0.8	0.1
Alkenylphosphatidylethanolamine (PE-P)	PE(P-36:2)	10.4	0.7
Alkenylphosphatidylethanolamine (PE-P)	PE(P-36:4)	19.9	0.5
Alkenylphosphatidylethanolamine (PE-P)	PE(P-38:4)	14.1	0.5
**Alkenylphosphatidylethanolamine (PE-P)**	**PE(P-38:5)**	**24.1**	**0.6**
Alkenylphosphatidylethanolamine (PE-P)	PE(P-38:6)	7.3	0.5
Alkenylphosphatidylethanolamine (PE-P)	PE(P-40:4)	0.9	0.1
Alkenylphosphatidylethanolamine (PE-P)	PE(P-40:5)	4.5	0.1
Alkenylphosphatidylethanolamine (PE-P)	PE(P-40:6)	3.0	0.2
Alkylphosphatidylcholine (PC-O)	PC(O-30:0)	0.1	0.0
Alkylphosphatidylcholine (PC-O)	PC(O-32:0)	1.0	0.0
Alkylphosphatidylcholine (PC-O)	PC(O-32:1)	0.4	0.0
Alkylphosphatidylcholine (PC-O)	PC(O-32:2)	0.1	0.0
Alkylphosphatidylcholine (PC-O)	PC(O-34:0)	0.2	0.0
Alkylphosphatidylcholine (PC-O)	PC(O-34:1)	5.3	0.3
Alkylphosphatidylcholine (PC-O)	PC(O-34:2)	22.7	0.7
Alkylphosphatidylcholine (PC-O)	PC(O-34:3)	0.5	0.0
Alkylphosphatidylcholine (PC-O)	PC(O-34:4)	0.8	0.1
Alkylphosphatidylcholine (PC-O)	PC(O-35:4)	0.5	0.0
Alkylphosphatidylcholine (PC-O)	PC(O-36:1)	0.4	0.0
Alkylphosphatidylcholine (PC-O)	PC(O-36:2)	2.3	0.1
Alkylphosphatidylcholine (PC-O)	PC(O-36:3)	9.2	0.2
Alkylphosphatidylcholine (PC-O)	**PC(O-36:4)**	**38.0**	**0.6**

Supplementary Table 1-Contd.

Class	Component	Mean percent of class	SEM
Alkylphosphatidylcholine (PC-O)	PC(O-36:5)	2.5	0.2
Alkylphosphatidylcholine (PC-O)	PC(O-38:4)	2.3	0.1
Alkylphosphatidylcholine (PC-O)	PC(O-38:5)	6.5	0.3
Alkylphosphatidylcholine (PC-O)	PC(O-40:6)	3.0	0.2
Alkylphosphatidylcholine (PC-O)	PC(O-40:7)	4.2	0.3
Alkylphosphatidylethanolamine (PE-O)	PE(O-34:1)	2.6	0.1
Alkylphosphatidylethanolamine (PE-O)	PE(O-34:2)	3.3	0.2
Alkylphosphatidylethanolamine (PE-O)	PE(O-36:2)	3.1	0.2
Alkylphosphatidylethanolamine (PE-O)	PE(O-36:3)	3.6	0.2
**Alkylphosphatidylethanolamine (PE-O)**	**PE(O-36:4)**	**31.8**	**1.0**
Alkylphosphatidylethanolamine (PE-O)	PE(O-36:5)	2.3	0.2
Alkylphosphatidylethanolamine (PE-O)	PE(O-36:6)	7.0	0.7
Alkylphosphatidylethanolamine (PE-O)	PE(O-38:4)	14.1	0.5
Alkylphosphatidylethanolamine (PE-O)	PE(O-38:5)	16.4	0.6
Alkylphosphatidylethanolamine (PE-O)	PE(O-40:5)	4.4	0.2
Alkylphosphatidylethanolamine (PE-O)	PE(O-40:6)	3.4	0.2
Alkylphosphatidylethanolamine (PE-O)	PE(O-40:7)	6.7	0.3
Alkylphosphatidylethanolamine (PE-O)	PE(O-42:7)	1.4	0.1
Ceramide (Cer)	Cer(d18:1/16:0)	4.4	0.1
Ceramide (Cer)	Cer(d18:1/18:0)	28.6	0.7
Ceramide (Cer)	Cer(d18:1/20:0)	1.4	0.1
Ceramide (Cer)	Cer(d18:1/22:0)	11.8	0.3
**Ceramide (Cer)**	**Cer(d18:1/24:0)**	**30.2**	**0.5**
Ceramide (Cer)	Cer(d18:1/24:1)	23.5	0.5
Cholesteryl ester (CE)	CE(14:0)	3.4	0.2
Cholesteryl ester (CE)	CE(15:0)	5.1	0.3
Cholesteryl ester (CE)	CE(16:0)	14.1	0.4
Cholesteryl ester (CE)	CE(16:1)	24.0	0.8
Cholesteryl ester (CE)	CE(17:1)	2.3	0.1
Cholesteryl ester (CE)	CE(18:0)	1.1	0.1
Cholesteryl ester (CE)	CE(18:1)	11.2	0.3
**Cholesteryl ester (CE)**	**CE(18:2)**	**31.2**	**1.1**
Cholesteryl ester (CE)	CE(18:3)	1.8	0.1
Cholesteryl ester (CE)	CE(20:3)	0.9	0.0
Cholesteryl ester (CE)	CE(20:4)	6.4	0.4
Diacylglycerol (DG)	DG(14:0_16:0)	1.8	0.1
Diacylglycerol (DG)	DG(14:0_18:1)	1.9	0.1
Diacylglycerol (DG)	DG(16:0_16:0)	4.0	0.2
Diacylglycerol (DG)	DG(16:0_18:0)	5.8	0.5
Diacylglycerol (DG)	DG(16:0_18:1)	17.8	0.4

Supplementary Table 1-Contd.

Class	Component	Mean percent of class	SEM
Diacylglycerol (DG)	DG(16:0_18:2)	6.7	0.3
Diacylglycerol (DG)	DG(16:0_22:5)	1.1	0.1
Diacylglycerol (DG)	DG(16:0_22:6)	1.0	0.1
Diacylglycerol (DG)	DG(16:1_18:1)	4.7	0.3
Diacylglycerol (DG)	DG(18:0_18:0)	3.5	0.6
Diacylglycerol (DG)	DG(18:0_18:1)	5.5	0.2
Diacylglycerol (DG)	DG(18:0_18:2)	4.6	0.1
**Diacylglycerol (DG)**	**DG(18:0_20:4)**	**18.1**	**0.6**
Diacylglycerol (DG)	DG(18:1_18:1)	14.1	0.4
Diacylglycerol (DG)	DG(18:1_18:2)	8.3	0.3
Diacylglycerol (DG)	DG(18:1_20:4)	1.2	0.1
Diacylglycerol (DG)	DG(18:2_18:2)	1.0	0.1
Dihexosylceramide (Hex2Cer)	Hex2Cer(d18:1/16:0)	12.2	0.7
**Dihexosylceramide (Hex2Cer)**	**Hex2Cer(d18:1/18:0)**	**45.3**	**0.9**
Dihexosylceramide (Hex2Cer)	Hex2Cer(d18:1/22:0)	13.5	0.5
Dihexosylceramide (Hex2Cer)	Hex2Cer(d18:1/24:0)	13.4	0.4
Dihexosylceramide (Hex2Cer)	Hex2Cer(d18:1/24:1)	15.7	0.5
**Dihydroceramide (dhCer)**	**Cer(d18:0/24:0)**	**100.0**	**0.0**
**Free cholesterol (COH)**	**COH**	**100.0**	**0.0**
**G_{M3} ganglioside (GM3)**	**GM3(d18:1/18:0)**	**54.8**	**1.2**
G_{M3} ganglioside (GM3)	GM3(d18:1/22:0)	12.1	0.7
G_{M3} ganglioside (GM3)	GM3(d18:1/24:0)	16.6	0.6
G_{M3} ganglioside (GM3)	GM3(d18:1/24:1)	16.7	0.7
Lysophosphatidylcholine (LPC)	LPC(14:0)	0.6	0.0
Lysophosphatidylcholine (LPC)	LPC(16:0)	9.9	0.9
Lysophosphatidylcholine (LPC)	LPC(16:1)	0.6	0.0
Lysophosphatidylcholine (LPC)	LPC(18:0)	3.8	0.3
Lysophosphatidylcholine (LPC)	LPC(18:1)	6.5	0.3
**Lysophosphatidylcholine (LPC)**	**LPC(18:2)**	**51.1**	**1.4**
Lysophosphatidylcholine (LPC)	LPC(18:3)	0.8	0.0
Lysophosphatidylcholine (LPC)	LPC(20:3)	3.8	0.1
Lysophosphatidylcholine (LPC)	LPC(20:4)	12.3	0.7
Lysophosphatidylcholine (LPC)	LPC(20:5)	2.3	0.3
Lysophosphatidylcholine (LPC)	LPC(22:5)	5.7	0.5
Lysophosphatidylcholine (LPC)	LPC(22:6)	2.7	0.2
Lysophosphatidylethanolamine (LPE)	LPE(18:0)	10.4	0.5
Lysophosphatidylethanolamine (LPE)	LPE(18:1)	5.6	0.6
Lysophosphatidylethanolamine (LPE)	LPE(20:2)	18.7	1.7
**Lysophosphatidylethanolamine (LPE)**	**LPE(20:4)**	**42.5**	**1.4**

Supplementary Table 1-Contd.

Class	Component	Mean percent of class	SEM
Lysophosphatidylethanolamine (LPE)	LPE(22:4)	6.2	0.4
Lysophosphatidylethanolamine (LPE)	LPE(22:5)	10.3	0.5
Lysophosphatidylethanolamine (LPE)	LPE(22:6)	12.4	0.9
**Monohexocylceramide (HexCer)**	**HexCer(d18:1/24:0)**	**100.0**	**0.0**
Phosphatidylcholine (PC)	PC(28:0)	0.1	0.0
Phosphatidylcholine (PC)	PC(29:0)	0.0	0.0
Phosphatidylcholine (PC)	PC(30:0)	0.4	0.0
Phosphatidylcholine (PC)	PC(31:0)	0.1	0.0
Phosphatidylcholine (PC)	PC(31:1)	0.1	0.0
Phosphatidylcholine (PC)	PC(32:0)	1.0	0.0
Phosphatidylcholine (PC)	PC(32:1)	2.1	0.1
Phosphatidylcholine (PC)	PC(32:2)	1.0	0.0
Phosphatidylcholine (PC)	PC(32:3)	0.0	0.0
Phosphatidylcholine (PC)	PC(33:0)	0.1	0.0
Phosphatidylcholine (PC)	PC(33:1)	0.6	0.0
Phosphatidylcholine (PC)	PC(33:2)	0.5	0.0
Phosphatidylcholine (PC)	PC(33:3)	0.0	0.0
Phosphatidylcholine (PC)	PC(34:0)	0.3	0.0
Phosphatidylcholine (PC)	PC(34:1)	13.9	0.3
**Phosphatidylcholine (PC)**	**PC(34:2)**	**42.0**	**0.7**
Phosphatidylcholine (PC)	PC(34:3)	1.5	0.0
Phosphatidylcholine (PC)	PC(34:4)	0.1	0.0
Phosphatidylcholine (PC)	PC(34:5)	0.0	0.0
Phosphatidylcholine (PC)	PC(35:0)	0.0	0.0
Phosphatidylcholine (PC)	PC(35:1)	0.4	0.0
Phosphatidylcholine (PC)	PC(35:2)	1.0	0.0
Phosphatidylcholine (PC)	PC(35:3)	0.1	0.0
Phosphatidylcholine (PC)	PC(35:4)	0.0	0.0
Phosphatidylcholine (PC)	PC(36:0)	0.0	0.0
Phosphatidylcholine (PC)	PC(36:1)	2.0	0.1
Phosphatidylcholine (PC)	PC(36:2)	13.8	0.3
Phosphatidylcholine (PC)	PC(36:3)	8.6	0.2
Phosphatidylcholine (PC)	PC(36:4)	0.5	0.0
Phosphatidylcholine (PC)	PC(36:4b)	3.3	0.1
Phosphatidylcholine (PC)	PC(36:5)	0.6	0.1
Phosphatidylcholine (PC)	PC(36:6)	0.0	0.0
Phosphatidylcholine (PC)	PC(37:4)	0.1	0.0
Phosphatidylcholine (PC)	PC(37:5)	0.0	0.0
Phosphatidylcholine (PC)	PC(38:2)	0.5	0.0
Phosphatidylcholine (PC)	PC(38:3)	0.6	0.0

Supplementary Table 1-Contd.

Class	Component	Mean percent of class	SEM
Phosphatidylcholine (PC)	PC(38:4)	1.2	0.1
Phosphatidylcholine (PC)	PC(38:5)	1.7	0.1
Phosphatidylcholine (PC)	PC(38:6a)	0.1	0.0
Phosphatidylcholine (PC)	PC(38:6b)	0.8	0.0
Phosphatidylcholine (PC)	PC(38:7)	0.0	0.0
Phosphatidylcholine (PC)	PC(39:6)	0.0	0.0
Phosphatidylcholine (PC)	PC(40:5)	0.3	0.0
Phosphatidylcholine (PC)	PC(40:6)	0.2	0.0
Phosphatidylcholine (PC)	PC(40:7)	0.1	0.0
Phosphatidylethanolamine (PE)	PE(32:0)	0.1	0.0
Phosphatidylethanolamine (PE)	PE(32:1)	0.1	0.0
Phosphatidylethanolamine (PE)	PE(34:1)	1.2	0.0
Phosphatidylethanolamine (PE)	PE(34:2)	1.8	0.0
Phosphatidylethanolamine (PE)	PE(34:3)	0.2	0.0
Phosphatidylethanolamine (PE)	PE(35:1)	0.1	0.0
Phosphatidylethanolamine (PE)	PE(35:2)	0.3	0.0
Phosphatidylethanolamine (PE)	PE(36:0)	0.1	0.0
Phosphatidylethanolamine (PE)	PE(36:1)	1.1	0.0
Phosphatidylethanolamine (PE)	PE(36:2)	18.0	0.5
Phosphatidylethanolamine (PE)	PE(36:3)	5.0	0.2
Phosphatidylethanolamine (PE)	PE(36:4)	1.8	0.0
Phosphatidylethanolamine (PE)	PE(36:5)	0.4	0.0
Phosphatidylethanolamine (PE)	PE(38:3)	6.1	0.1
**Phosphatidylethanolamine (PE)**	**PE(38:4)**	**42.6**	**0.8**
Phosphatidylethanolamine (PE)	PE(38:5)	8.0	0.3
Phosphatidylethanolamine (PE)	PE(38:6)	1.3	0.1
Phosphatidylethanolamine (PE)	PE(40:5)	3.0	0.1
Phosphatidylethanolamine (PE)	PE(40:6)	7.8	0.5
Phosphatidylethanolamine (PE)	PE(40:7)	1.0	0.1
**Phosphatidylglycerol (PG)**	**PG(34:1)**	**58.5**	**0.9**
Phosphatidylglycerol (PG)	PG(34:2)	5.7	0.3
Phosphatidylglycerol (PG)	PG(36:1)	25.7	1.0
Phosphatidylglycerol (PG)	PG(36:2)	10.1	0.4
Phosphatidylinositol (PI)	PI(32:0)	0.2	0.0
Phosphatidylinositol (PI)	PI(32:1)	0.1	0.0
Phosphatidylinositol (PI)	PI(34:0)	0.9	0.1
Phosphatidylinositol (PI)	PI(34:1)	1.5	0.1
Phosphatidylinositol (PI)	PI(36:1)	2.7	0.2
Phosphatidylinositol (PI)	PI(36:2)	13.0	0.4
Phosphatidylinositol (PI)	PI(36:3)	1.4	0.0

Authors: Tonks, Coster, Christopher, Chaudhuri, Xu, Gagnon-Bartsch, Chisholm, James, Meikle, Greenfield and Samocha-Bonet
Contact: peter.meikle@bakeridi.edu.au or d.samochabonet@garvan.org.au

Supplementary Table 1-Contd.

Class	Component	Mean percent of class	SEM
Phosphatidylinositol (PI)	PI(36:4)	0.4	0.0
Phosphatidylinositol (PI)	PI(38:2)	1.1	0.1
Phosphatidylinositol (PI)	PI(38:3)	17.0	0.4
**Phosphatidylinositol (PI)**	**PI(38:4)**	**55.2**	**0.7**
Phosphatidylinositol (PI)	PI(38:5)	2.1	0.1
Phosphatidylinositol (PI)	PI(40:4)	1.2	0.0
Phosphatidylinositol (PI)	PI(40:5)	2.0	0.1
Phosphatidylinositol (PI)	PI(40:6)	1.3	0.1
Phosphatidylserine (PS)	PS(36:1)	13.4	0.4
Phosphatidylserine (PS)	PS(36:2)	23.2	1.0
Phosphatidylserine (PS)	PS(38:3)	4.7	0.2
Phosphatidylserine (PS)	PS(38:4)	10.0	0.4
Phosphatidylserine (PS)	PS(38:5)	1.3	0.1
Phosphatidylserine (PS)	PS(40:5)	18.3	0.4
**Phosphatidylserine (PS)**	**PS(40:6)**	**29.1**	**1.2**
Sphingomyelin (SM)	SM(31:1)	0.1	0.0
Sphingomyelin (SM)	SM(32:1)	0.4	0.0
Sphingomyelin (SM)	SM(32:2)	0.0	0.0
Sphingomyelin (SM)	SM(33:1)	0.3	0.0
Sphingomyelin (SM)	SM(34:1)	4.8	0.2
Sphingomyelin (SM)	SM(34:2)	0.4	0.0
Sphingomyelin (SM)	SM(35:1)	0.7	0.0
Sphingomyelin (SM)	SM(35:2)	0.0	0.0
Sphingomyelin (SM)	SM(36:1)	6.3	0.2
Sphingomyelin (SM)	SM(36:2)	1.0	0.0
Sphingomyelin (SM)	SM(36:3)	0.0	0.0
**Sphingomyelin (SM)**	**SM(38:1)**	**78.7**	**0.5**
Sphingomyelin (SM)	SM(38:2)	3.0	0.1
Sphingomyelin (SM)	SM(39:1)	0.5	0.0
Sphingomyelin (SM)	SM(41:1)	0.9	0.0
Sphingomyelin (SM)	SM(41:2)	0.5	0.0
Sphingomyelin (SM)	SM(42:1)	2.2	0.1
**Sphingosine (SPH)**	**SPH(d18:1)**	**100.0**	**0.0**
Triacylglycerol (TG)	TG(14:0_16:0_18:1)	3.6	0.1
Triacylglycerol (TG)	TG(14:0_16:0_18:2)	2.0	0.1
Triacylglycerol (TG)	TG(14:0_16:1_18:1)	3.0	0.1
Triacylglycerol (TG)	TG(14:0_16:1_18:2)	0.6	0.0
Triacylglycerol (TG)	TG(14:0_17:0_18:1)	0.8	0.0
Triacylglycerol (TG)	TG(14:0_18:0_18:1)	0.4	0.0
Triacylglycerol (TG)	TG(14:0_18:2_18:2)	0.2	0.0

Supplementary Table 1-Contd.

Class	Component	Mean percent of class	SEM
Triacylglycerol (TG)	TG(14:1_16:0_18:1)	0.7	0.0
Triacylglycerol (TG)	TG(14:1_16:1_18:0)	2.1	0.1
Triacylglycerol (TG)	TG(14:1_18:0_18:2)	0.3	0.0
Triacylglycerol (TG)	TG(14:1_18:1_18:1)	1.6	0.1
Triacylglycerol (TG)	TG(15:0_16:0_18:1)	0.5	0.0
Triacylglycerol (TG)	TG(15:0_18:1_18:1)	0.3	0.0
Triacylglycerol (TG)	TG(16:0_16:0_16:0)	1.4	0.1
Triacylglycerol (TG)	TG(16:0_16:0_18:0)	1.9	0.1
Triacylglycerol (TG)	TG(16:0_16:0_18:1)	8.6	0.3
Triacylglycerol (TG)	TG(16:0_16:0_18:2)	1.8	0.1
Triacylglycerol (TG)	TG(16:0_16:1_17:0)	0.8	0.0
Triacylglycerol (TG)	TG(16:0_16:1_18:1)	10.3	0.3
Triacylglycerol (TG)	TG(16:0_17:0_18:1)	0.6	0.0
Triacylglycerol (TG)	TG(16:0_17:0_18:2)	0.7	0.0
Triacylglycerol (TG)	TG(16:0_18:0_18:1)	5.4	0.3
**Triacylglycerol (TG)**	**TG(16:0_18:1_18:1)**	**17.2**	**0.3**
Triacylglycerol (TG)	TG(16:0_18:1_18:2)	7.4	0.2
Triacylglycerol (TG)	TG(16:0_18:2_18:2)	1.5	0.1
Triacylglycerol (TG)	TG(16:1_16:1_16:1)	0.3	0.0
Triacylglycerol (TG)	TG(16:1_16:1_18:0)	0.5	0.0
Triacylglycerol (TG)	TG(16:1_16:1_18:1)	1.5	0.1
Triacylglycerol (TG)	TG(16:1_17:0_18:1)	1.4	0.0
Triacylglycerol (TG)	TG(16:1_18:1_18:1)	2.5	0.1
Triacylglycerol (TG)	TG(16:1_18:1_18:2)	2.4	0.1
Triacylglycerol (TG)	TG(17:0_18:1_18:1)	0.6	0.0
Triacylglycerol (TG)	TG(18:0_18:0_18:0)	0.1	0.0
Triacylglycerol (TG)	TG(18:0_18:0_18:1)	0.4	0.0
Triacylglycerol (TG)	TG(18:0_18:1_18:1)	4.2	0.1
Triacylglycerol (TG)	TG(18:0_18:2_18:2)	0.3	0.0
Triacylglycerol (TG)	TG(18:1_18:1_18:1)	7.2	0.2
Triacylglycerol (TG)	TG(18:1_18:1_18:2)	3.2	0.1
Triacylglycerol (TG)	TG(18:1_18:1_20:4)	0.2	0.0
Triacylglycerol (TG)	TG(18:1_18:1_22:6)	0.2	0.0
Triacylglycerol (TG)	TG(18:1_18:2_18:2)	1.5	0.1
Triacylglycerol (TG)	TG(18:2_18:2_18:2)	0.1	0.0
Trihexosylceramide (Hex3Cer)	Hex3Cer(d18:1/16:0)	13.6	0.7
Trihexosylceramide (Hex3Cer)	Hex3Cer(d18:1/18:0)	19.9	1.4
Trihexosylceramide (Hex3Cer)	Hex3Cer(d18:1/22:0)	21.6	0.9
**Trihexosylceramide (Hex3Cer)**	**Hex3Cer(d18:1/24:0)**	**29.8**	**1.0**
Trihexosylceramide (Hex3Cer)	Hex3Cer(d18:1/24:1)	16.0	0.6

Authors: Tonks, Coster, Christopher, Chaudhuri, Xu, Gagnon-Bartsch, Chisholm, James, Meikle, Greenfield and Samocha-Bonet
Contact: peter.meikle@bakeridi.edu.au or d.samochabonet@garvan.org.au

Supplementary Table 2: Characteristics of the gene array cohort

	Lean (n=13)	Overweight/Obese		ANOVA P-value
		Insulin-sensitive (OIS, n=11)	Insulin-resistant (OIR, n=11)	
Age (years)	57 ± 2	60 ± 10	55 ± 2	0.4
BMI (kg/m^2)	21.5 ± 0.5	29.5 ± 1.2**	33.8 ± 2.4**	<0.001
Body fat (%)	27 ± 2	42 ± 2**	38 ± 3**	0.001
L2/L3 Visceral area (cm^2)	46 ± 8	143 ± 50	270 ± 25**#	<0.001
L2/L3 Subcutaneous area (cm^2)	78 ± 13	227 ± 30**	233 ± 48**	0.02
CT attenuation Liver/Spleen ratio	1.29 ± 0.11	1.27 ± 0.15	0.94 ± 0.09	0.08
Systolic blood pressure (mm Hg)	118 ± 4	129 ± 5	134 ± 5	0.06
Diastolic blood pressure (mm Hg)	72 ± 2	83 ± 3*	85 ± 2**	<0.001
Glucose infusion rate (µmol/min/kg FFM)	95 ± 8	97 ± 13	49 ± 4**##	<0.001
$RQ_{Baseline}$	0.80 ± 0.01	0.82 ± 0.01	0.83 ± 0.02	0.4
Δ RQ ($RQ_{Baseline}$-RQ_{Clamp})	0.14 ± 0.01	0.11 ± 0.04	0.05 ± 0.02**#	<0.001
Fasting blood glucose (mmol/L)	4.5 ± 0.1	4.7 ± 0.1	5.3 ± 0.1**##	<0.001
Fasting serum insulin (mU/L)	12 ± 1	12 ± 2	22 ± 2**##	<0.001
HOMA-IR	1.0 ± 0.1	1.3 ± 0.1	4.7 ± 0.3**##	<0.001

Data are mean ± SEM

Differences *vs*. the Lean group *P<0.05 and **P<0.01 and *vs*. the OIS group #P<0.05 and ##P<0.01 by one-way ANOVA and Tukey posthoc analyses.

Skeletal muscle and plasma lipidomic signatures of insulin resistance and overweight/obesity in humans

Authors: Tonks, Coster, Christopher, Chaudhuri, Xu, Gagnon-Bartsch, Chisholm, James, Meikle, Greenfield and Samocha-Bonet
Contact: peter.meikle@bakeridi.edu.au or d.samochabonet@garvan.org.au

Supplementary Table 3: KEGG pathways that were significantly up- or down- regulated between the groups

**Pathway**	**OIR *vs* Lean**	**OIR *vs* OIS**	**OIS *vs* Lean**	**Legend**
KEGG_ERBB_SIGNALING_PATHWAY	3	2	1	3 Mixed
KEGG_GLIOMA	3	2	1	2 Down
KEGG_VALINE_LEUCINE_AND_ISOLEUCINE_DEGRADATION	2	3	0	1 Up
KEGG_DRUG_METABOLISM_OTHER_ENZYMES	2	2	0	
KEGG_DRUG_METABOLISM_CYTOCHROME_P450	2	2	0	
KEGG_GLYCOSAMINOGLYCAN_DEGRADATION	2	2	0	
KEGG_STEROID_HORMONE_BIOSYNTHESIS	2	2	0	
KEGG_GLUTATHIONE_METABOLISM	2	2	0	
KEGG_INSULIN_SIGNALING_PATHWAY	3	2	0	
KEGG_VALINE_LEUCINE_AND_ISOLEUCINE_BIOSYNTHESIS	3	3	0	
KEGG_ARGININE_AND_PROLINE_METABOLISM	3	3	0	
KEGG_PROSTATE_CANCER	3	0	1	
KEGG_HUNTINGTONS_DISEASE	2	0	2	
KEGG_PARKINSONS_DISEASE	2	0	2	
KEGG_ALZHEIMERS_DISEASE	2	0	2	
KEGG_MATURITY_ONSET_DIABETES_OF_THE_YOUNG	2	0	2	
KEGG_CARDIAC_MUSCLE_CONTRACTION	2	0	2	
KEGG_NEUROACTIVE_LIGAND_RECEPTOR_INTERACTION	2	0	2	
KEGG_OXIDATIVE_PHOSPHORYLATION	2	0	2	
KEGG_LINOLEIC_ACID_METABOLISM	2	0	2	
KEGG_PROTEIN_EXPORT	3	1	0	
KEGG_SMALL_CELL_LUNG_CANCER	3	0	0	
KEGG_GLYCINE_SERINE_AND_THREONINE_METABOLISM	3	0	0	
KEGG_LYSOSOME	3	0	0	
KEGG_TASTE_TRANSDUCTION	2	0	0	
KEGG_OLFACTORY_TRANSDUCTION	2	0	0	
KEGG_VEGF_SIGNALING_PATHWAY	2	0	0	
KEGG_ABC_TRANSPORTERS	2	0	0	
KEGG_ARACHIDONIC_ACID_METABOLISM	2	0	0	
KEGG_ALPHA_LINOLENIC_ACID_METABOLISM	2	0	0	
KEGG_PENTOSE_AND_GLUCURONATE_INTERCONVERSIONS	2	2	3	
KEGG_RIBOSOME	2	2	2	
KEGG_COMPLEMENT_AND_COAGULATION_CASCADES	0	1	2	
KEGG_ARRHYTHMOGENIC_RIGHT_VENTRICULAR_CARDIOMYOPATHY_ARVC	0	1	2	
KEGG_CELL_ADHESION_MOLECULES_CAMS	0	0	3	
KEGG_LIMONENE_AND_PINENE_DEGRADATION	0	0	3	
KEGG_PORPHYRIN_AND_CHLOROPHYLL_METABOLISM	0	0	3	
KEGG_STARCH_AND_SUCROSE_METABOLISM	0	0	3	

Supplementary Table 3-Contd.

**Pathway**	**OIR *vs* Lean**	**OIR *vs* OIS**	**OIS *vs* Lean**	**Legend**
KEGG_ASCORBATE_AND_ALDARATE_METABOLISM	0	0	3	
KEGG_TYROSINE_METABOLISM	0	0	3	
KEGG_DILATED_CARDIOMYOPATHY	0	0	2	
KEGG_OTHER_GLYCAN_DEGRADATION	0	0	2	3 Mixed
KEGG_HYPERTROPHIC_CARDIOMYOPATHY_HCM	0	0	2	2 Down
KEGG_GRAFT_VERSUS_HOST_DISEASE	0	0	1	1 Up
KEGG_ENDOMETRIAL_CANCER	0	0	1	
KEGG_PANCREATIC_CANCER	0	0	1	
KEGG_COLORECTAL_CANCER	0	0	1	
KEGG_VASOPRESSIN_REGULATED_WATER_REABSORPTION	0	0	1	
KEGG_MELANOGENESIS	0	0	1	
KEGG_PROGESTERONE_MEDIATED_OOCYTE_MATURATION	0	0	1	
KEGG_T_CELL_RECEPTOR_SIGNALING_PATHWAY	0	0	1	
KEGG_NATURAL_KILLER_CELL_MEDIATED_CYTOTOXICITY	0	0	1	
KEGG_TGF_BETA_SIGNALING_PATHWAY	0	0	1	
KEGG_OOCYTE_MEIOSIS	0	0	1	
KEGG_CELL_CYCLE	0	0	1	
KEGG_BASAL_TRANSCRIPTION_FACTORS	0	0	1	
KEGG_SPLICEOSOME	0	0	1	
KEGG_BASAL_CELL_CARCINOMA	1	0	1	
KEGG_PATHWAYS_IN_CANCER	1	0	1	
KEGG_ANTIGEN_PROCESSING_AND_PRESENTATION	1	0	1	
KEGG_PHOSPHATIDYLINOSITOL_SIGNALING_SYSTEM	1	0	1	
KEGG_UBIQUITIN_MEDIATED_PROTEOLYSIS	1	0	1	
KEGG_SYSTEMIC_LUPUS_ERYTHEMATOSUS	1	1	1	
KEGG_EPITHELIAL_CELL_SIGNALING_IN_HELICOBACTER_PYLORI_INFECTION	0	1	0	
KEGG_VIBRIO_CHOLERAE_INFECTION	0	1	0	
KEGG_O_GLYCAN_BIOSYNTHESIS	0	1	0	
KEGG_NICOTINATE_AND_NICOTINAMIDE_METABOLISM	0	1	0	
KEGG_PROTEIN_DIGESTION_AND_ABSORPTION	1	1	0	
KEGG_PATHOGENIC_ESCHERICHIA_COLI_INFECTION	1	1	0	
KEGG_REGULATION_OF_ACTIN_CYTOSKELETON	1	1	0	
KEGG_ECM_RECEPTOR_INTERACTION	1	1	0	
KEGG_FOCAL_ADHESION	1	1	0	
KEGG_ENDOCYTOSIS	1	1	0	
KEGG_PROTEASOME	1	1	0	
KEGG_RIBOFLAVIN_METABOLISM	1	1	0	
KEGG_GLYCOLYSIS_GLUCONEOGENESIS	1	1	0	
KEGG_SPHINGOLIPID_METABOLISM	1	1	0	

Supplementary Table 3-Contd.

Pathway	OIR *vs* Lean	OIR *vs* OIS	OIS *vs* Lean	Legend	
KEGG_STAPHYLOCOCCUS_AUREUS_INFECTION	1	0	0		
KEGG_VIRAL_MYOCARDITIS	1	0	0		
KEGG_FC_GAMMA_R_MEDIATED_PHAGOCYTOSIS	1	0	0		
KEGG_TOLL_LIKE_RECEPTOR_SIGNALING_PATHWAY	1	0	0		
KEGG_ADHERENS_JUNCTION	1	0	0		
KEGG_WNT_SIGNALING_PATHWAY	1	0	0	3	Mixed
KEGG_P53_SIGNALING_PATHWAY	1	0	0	2	Down
KEGG_FRUCTOSE_AND_MANNOSE_METABOLISM	1	0	0	1	Up
KEGG_N_GLYCAN_BIOSYNTHESIS	1	0	0		
KEGG_APOPTOSIS	1	3	0		
KEGG_ALLOGRAFT_REJECTION	0	2	1		
KEGG_AUTOIMMUNE_THYROID_DISEASE	0	2	1		
KEGG_NON_SMALL_CELL_LUNG_CANCER	0	2	1		
KEGG_CHRONIC_MYELOID_LEUKEMIA	0	2	1		
KEGG_RENAL_CELL_CARCINOMA	0	2	1		
KEGG_NEUROTROPHIN_SIGNALING_PATHWAY	0	2	1		
KEGG_CIRCADIAN_RHYTHM_MAMMAL	0	2	1		
KEGG_NOTCH_SIGNALING_PATHWAY	0	2	1		
KEGG_MISMATCH_REPAIR	0	2	1		
KEGG_NUCLEOTIDE_EXCISION_REPAIR	0	2	1		
KEGG_ONE_CARBON_POOL_BY_FOLATE	0	2	1		
KEGG_DNA_REPLICATION	0	2	1		
KEGG_BLADDER_CANCER	0	2	0		
KEGG_LONG_TERM_POTENTIATION	0	2	0		
KEGG_HOMOLOGOUS_RECOMBINATION	0	2	0		
KEGG_AMINOACYL_TRNA_BIOSYNTHESIS	0	2	0		
KEGG_NITROGEN_METABOLISM	0	2	0		
KEGG_ALANINE_ASPARTATE_AND_GLUTAMATE_METABOLISM	0	2	0		
KEGG_STEROID_BIOSYNTHESIS	0	2	0		
KEGG_PYRIMIDINE_METABOLISM	0	2	0		
KEGG_ACUTE_MYELOID_LEUKEMIA	0	3	0		
KEGG_PROPANOATE_METABOLISM	0	3	0		
KEGG_PYRUVATE_METABOLISM	0	3	0		
KEGG_SELENOAMINO_ACID_METABOLISM	0	3	0		
KEGG_CYSTEINE_AND_METHIONINE_METABOLISM	0	3	0		
KEGG_BETA_ALANINE_METABOLISM	0	3	0		

Skeletal muscle and plasma lipidomic signatures of insulin resistance and overweight/obesity in humans

Authors: Tonks, Coster, Christopher, Chaudhuri, Xu, Gagnon-Bartsch, Chisholm, James, Meikle, Greenfield and Samocha-Bonet
Contact: peter.meikle@bakeridi.edu.au or d.samochabonet@garvan.org.au

Supplementary Figure 1A:

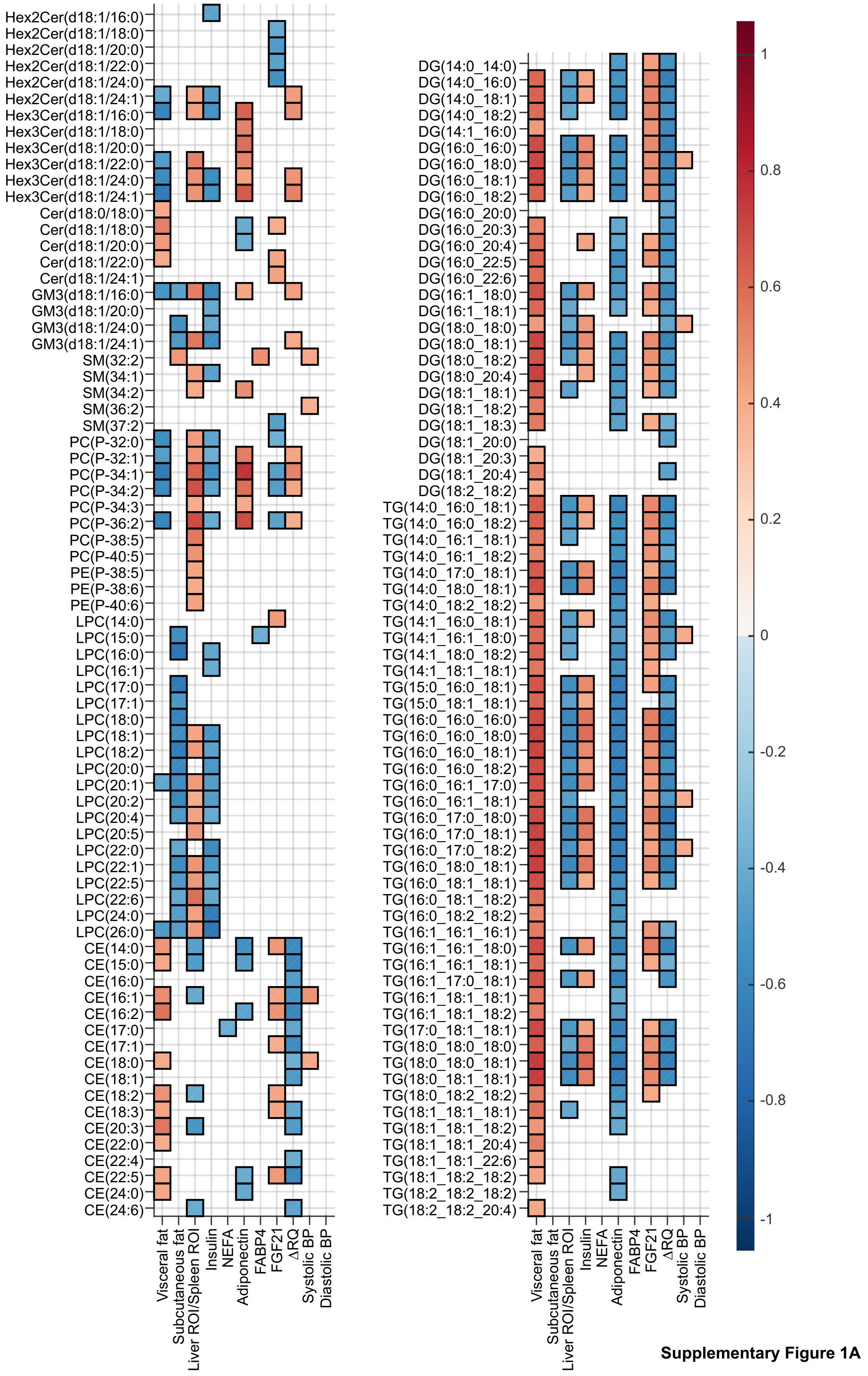

Skeletal muscle and plasma lipidomic signatures of insulin resistance and overweight/obesity in humans

Authors: Tonks, Coster, Christopher, Chaudhuri, Xu, Gagnon-Bartsch, Chisholm, James, Meikle, Greenfield and Samocha-Bonet
Contact: peter.meikle@bakeridi.edu.au or d.samochabonet@garvan.org.au

Supplementary Figure 1B:

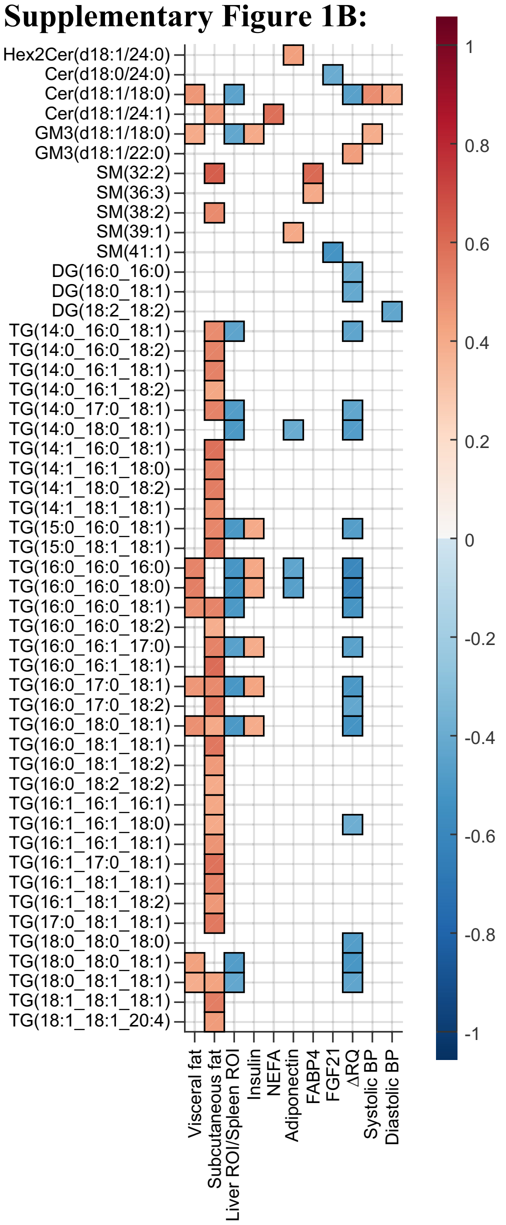

Supplementary Figure 1B

Supplementary Figure 1: Heat map based on Pearson's correlations between plasma (A) and skeletal muscle (B) lipids with abdominal fat distribution (at the L2/L3 slice), liver-to-spleen attenuation ratio (inverse marker of liver fat), circulating fasting insulin, non-esterified fatty acids (NEFA), cytokines, metabolic flexibility (ΔRQ) and blood pressure. Lipids included in both muscle and plasma correlations were sphingolipids, DG and TG. Additional lipids considered in plasma were CE, plasmalogens (PE(P) and PC(P)) and LPC. The *R* value of the correlation is shown in the color and only correlations with P<0.01 are presented.
